# JMJD3-driven epigenetic reprogramming of p16^INK4a^-positive cells promotes tendon regeneration

**DOI:** 10.1038/s41413-026-00537-1

**Published:** 2026-06-11

**Authors:** Xiaonan Liu, Wenhao Lu, Yicheng Wang, Peilin Zhang, Zhongyi Su, Ting Cao, Yifei Zhang, Junjie Zhao, Jiayi Wang, Hulin Xiang, Weijun Huang, Kai Wang, Sa Pang, Minkai Chen, Yusheng Li, Shen Liu

**Affiliations:** 1 Department of Orthopaedics, Shanghai Sixth People’s Hospital Affiliated to Shanghai Jiao Tong University School of Medicine, Shanghai, PR China; 2 Department of Orthopedics, Xiangya Hospital, Central South University, Changsha, Hunan PR China; 3 National Clinical Research Center for Geriatric Disorders (Xiangya Hospital), Central South University, Changsha, Hunan PR China; 4 Hainan Academy of Medical Sciences, Hainan Medical University, Haikou, Hainan PR China

**Keywords:** Pathogenesis, Diseases

## Abstract

Tendon healing is a complex biological process that involves inflammation, cellular reprogramming, and extracellular matrix (ECM) remodeling. The cyclin-dependent kinase inhibitor p16^INK4a^ is a well-characterized tumor suppressor associated with cell-cycle regulation under conditions of cellular stress and senescence. Although genetic ablation of p16^INK4a+^ cells has shown beneficial effects in age-associated tendinopathies, the cellular identity and functional contribution of p16^INK4a+^ cells during tendon regeneration remain poorly understood. In this study, we demonstrate that an injury-induced accumulation of p16^INK4a+^ cells in a murine Achilles tendon injury model promotes tendon structural and functional recovery. Using a murine tendon puncture model combined with single-cell RNA sequencing and in situ immunostaining, we identify these p16^INK4a+^ cells as predominantly mesenchymal cells characterized by elevated ECM gene expression. Mechanistically, p16^INK4a+^ cells exhibit increased activity of the histone demethylase JMJD3 (KDM6B), accompanied by reduced H3K27me3 levels and enhanced expression of ECM-related genes, including *Col1a1* and *Col3a1*, thus supporting organized collagen deposition and tissue remodeling. Genetic deletion or pharmacological inhibition of JMJD3 resulted in increased H3K27me3 levels, disrupted collagen organization, and reduced biomechanical strength. Conversely, pharmacological reduction of H3K27me3 was associated with enhanced ECM gene expression and accelerated tendon repair. Together, these findings identify a JMJD3-associated epigenetic program in p16^INK4a+^ cells that contributes to ECM remodeling during tendon healing, highlighting the beneficial and context-dependent role of p16^INK4a+^ cells in musculoskeletal tissue repair.

## Introduction

Tendon injury is a common musculoskeletal condition that frequently results in incomplete healing and persistent functional impairment due to the limited intrinsic regenerative capacity of tendon tissue. The repair process following tendon injury involves a delicate interplay among inflammation, cellular differentiation, and extracellular matrix (ECM) remodeling.^1,2^ However, unlike other connective tissues, tendons tend to heal by forming fibrotic scar tissue rather than regenerating their native hierarchical structure, leading to reduced elasticity and a high risk of re-rupture.^3,4^ Despite advances in surgical techniques and rehabilitation strategies, the biological mechanisms governing successful tendon repair remain incompletely understood, highlighting the need to elucidate endogenous cellular programs that promote functional regeneration.

Cells expressing the cyclin-dependent kinase inhibitor p16^INK4a^ have traditionally been viewed as stressed cells and investigated in the context of cellular senescence and age-related tissue dysfunction, as p16^INK4a^ expression frequently increases in aged or chronically injured tissues.^5–7^ In these settings, p16^INK4a+^ populations have been associated with fibrosis and persistent inflammation through deleterious secretion, such as senescence-associated secretory phenotype (SASP) factors.^8–10^ However, accumulating evidence from acute injury models indicates that p16^INK4a+^ cells, although they may not meet the full criteria of classical senescence, can also emerge transiently during early tissue repair and participate in regenerative processes. For instance, p16^INK4a+^ cells have been implicated in skin repair, where they could secrete factors such as PDGF-AA and act as transient signaling for regeneration.^11^ Notably, a recent work demonstrated that p16^INK4a+^ cells arising in normal lung tissue can contribute to tissue remodeling in a context-dependent manner, further supporting the idea that p16^INK4a^ expression does not uniformly signify detrimental cellular states but may reflect dynamic responses to injury.^12^ Despite these advances, whether p16^INK4a+^ cells arise during tendon injury, which cell types they represent, and how they influence tendon repair remain unexplored.

Epigenetic regulation plays a critical role in orchestrating gene expression programs associated with both cell-cycle regulation and tissue regeneration.^13–15^ Among various epigenetic mechanisms, the trimethylation of histone H3 at lysine 27 (H3K27me3) is a key repressive mark deposited by the polycomb repressive complex 2 (PRC2) through its catalytic subunit EZH2, and its removal is mediated by histone demethylases such as UTX(KDM6A) and JMJD3 (KDM6B).^16,17^ JMJD3 has been reported to promote lineage-specific transcriptional activation during development and repair by erasing H3K27me3 at target gene loci.^18^ Importantly, JMJD3-driven chromatin remodeling was shown to be beneficial in regulating extracellular matrix organization, inflammation resolution, and progenitor differentiation.^19–21^ However, the mechanism linking p16^INK4a+^ cells, JMJD3-mediated H3K27 trimethylation, and tendon regeneration remains poorly characterized.

In this study, we employed a murine Achilles tendon puncture model to investigate the functional role of p16^INK4a+^ cells in tendon repair. We observed a transient accumulation of p16^INK4a+^ cells with senescence-associated characteristics at the injury site during tendon healing. Contrary to the conventional view of p16^INK4a+^ as a deleterious process, depletion of these cells impaired tendon regeneration, resulting in disorganized collagen fibers and reduced biomechanical strength. Mechanistically, we identified that p16^INK4a+^ cells exhibited pronounced upregulation of JMJD3, leading to the demethylation of H3K27me3 and subsequent activation of ECM-related genes, including *Col1a1* and *Col3a1*, which collectively promote collagen synthesis and matrix remodeling. Both genetic and pharmacological inhibition of JMJD3 abolished these beneficial effects, underscoring the pivotal role of JMJD3-mediated epigenetic remodeling in orchestrating senescence-associated pro-repair programs during tendon healing.

## Results

### p16^INK4a+^ cells promote tendon healing in mice

**Fig. 1:**
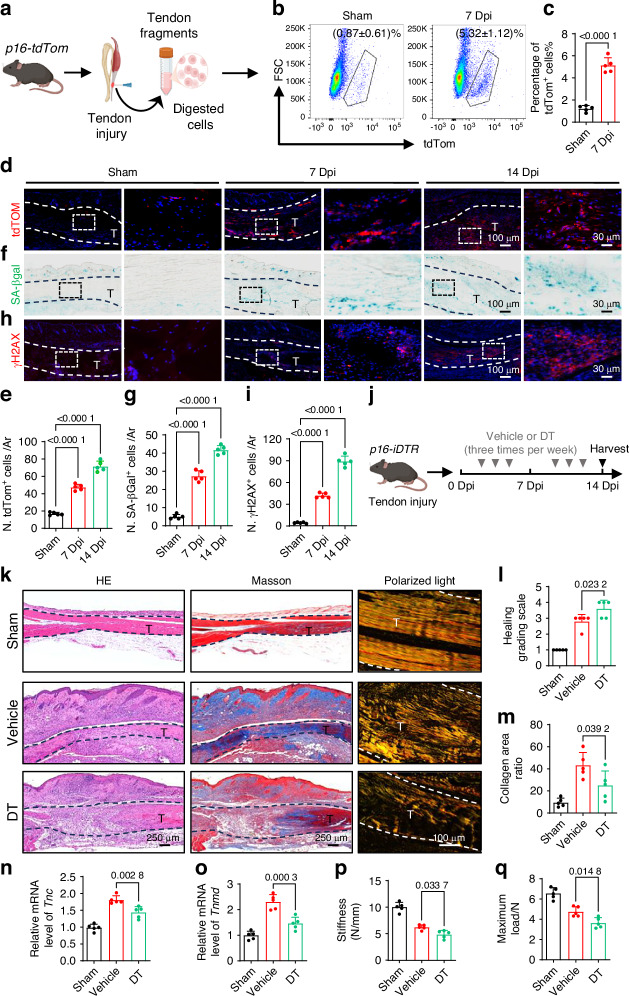
p16^INK4a+^ cells promote tendon healing in mice. Eight-week-old male *p16-tdTOM* mice were subjected to a central puncture in the middle of the right Achilles tendon and kept for one or two weeks until sacrifice. **a** Schematic diagram illustrating the experimental procedure. **b**, **c** Tendon tissues were enzymatically digested, and isolated cells were analyzed by flow cytometry. Representative flow cytometry plots showing tdTomato-positive (tdTom^+^) cells in sham and injured tendon tissues are shown in (**b**). Quantification of the percentage of tdTom^+^ cells in sham and injured tendon tissues is shown in (**c**). **d**, **e** Representative fluorescence images of tendon sections from sham and injured tendon mice at 7 days post-injury (7 Dpi) and 14 days post-injury (14 Dpi) are shown in (**d**). tdTomato fluorescence (red) labels p16^INK4a+^ cells, and nuclei are counterstained with DAPI (blue). Boxed regions are shown at higher magnification in the corresponding right panels. Quantification of tdTom^+^ cells per mm² of tendon tissue area is shown in (**e**). **f**, **g** Senescence-associated β-galactosidase (SA-βgal) staining was performed on tendon sections. Representative images of SA-βgal-positive cells (green) in sham and injured tendons are shown in (**f**). Boxed regions are shown at higher magnification in the corresponding right panels. Quantification of SA-βgal^+^ cells per mm² of tendon tissue area is shown in (**g**). **h**, **i** Immunofluorescence staining was performed on tendon sections using an antibody against γH2AX. Representative images of γH2AX^+^ cells (red) in sham and injured tendons are shown in (**h**). Nuclei are counterstained with DAPI (blue). Boxed regions are shown at higher magnification in the corresponding right panels. Quantification of γH2AX^+^ cells per mm² of tendon tissue area is shown in (**i**). **j** Schematic illustration of experimental design. Eight-week-old male *p16-iDTR* mice were subjected to a central puncture injury in the middle of the right Achilles tendon and treated with vehicle or diphtheria toxin (DT) to selectively ablate p16^INK4a+^ cells. Mice were sacrificed at 14 Dpi. **k**–**m** Representative images of tendon sections stained with hematoxylin and eosin (H&E), Masson’s trichrome, and picrosirius red under polarized light microscopy (PSR–PLM) are shown in (**k**). Quantification of the tendon healing grading scale is shown in (**l**) and the collagen area ratio is shown in (**m**). **n**, **o** Relative mRNA expression levels of tendon repair genes in healing tendons were assessed by qRT–PCR. Expression levels of *Tnc* and *Tnmd* are shown in (**n**) and (**o**) respectively. **p**, **q** Biomechanical properties of healing tendons were evaluated using a loading rate of 1 mm/min. Tendon stiffness is shown in (**p**), and maximum load to failure is shown in (**q**). T, tendon area. *n* = 5 mice per group. Data are presented as mean ± SD. Statistical significance was determined by one-way ANOVA, except for (**c**) which was analyzed using an unpaired two-tailed Student’s *t*-test

To investigate the role of p16^INK4a+^ cells in tendon healing, we employed *p16-Cre*^*ERT2*^*:: tdTomato* reporter mice (*p16-tdTOM* mice) to visualize and quantify the dynamics of p16^INK4a+^ cells following Achilles tendon injury (Fig. 1a). Mice were subjected to a standardized needle-puncture model, and Achilles tendons were collected at multiple time points post-injury (Dpi).^22^ Flow cytometry analysis of dissociated tendon tissues of *p16-tdTOM* mice confirmed the accumulation of p16^INK4a+^ cells, showing that tdTom^+^ cells increased from 0.87% ± 0.61% in the sham group to 5.32% ± 1.12% in the 7 Dpi group (Fig. 1b, c). Fluorescence imaging of tdTom revealed a gradual increase in tdTOM^+^ cells within the tendon lesion area (Fig. 1d, e). To assess whether p16^INK4a+^ cells exhibited senescence-associated features, we performed senescence-associated β-galactosidase (SA-β-gal) staining and immunofluorescence for the DNA damage marker γH2AX. The injured tendon exhibited strong SA-β-gal positivity (Fig. 1f, g) and elevated γH2AX foci staining (Fig. 1h, i). Quantitative analysis showed a 5.48-fold increase in SA-β-gal-positive cells and a 9.95-fold increase in γH2AX^+^ nuclei in the 7 Dpi group compared with the sham group. To determine the functional role of these p16^INK4a+^ cells during tendon healing, we generated *p16-Cre*^*ERT2*^*::iDTR* mice (*p16-iDTR* mice) to enable selective ablation of p16^INK4a+^ cells through diphtheria toxin (DT) administration (Fig. 1j). DT was given during the early-middle phase of repair (prior to 14 Dpi) and resulted in efficient depletion of p16^INK4a+^ cells, accompanied by reduced SA-β-gal staining (Fig. S1A, B). Histological evaluation by H&E staining revealed that p16^INK4a+^ cell depletion markedly impaired tendon healing on healing grading scale assessment (Fig. S2A–D). Masson’s trichrome staining demonstrated disorganized collagen fibers, reduced mesenchymal cellularity, and persistent inflammatory infiltration of DT-treated mice compared to vehicle-treated littermates (Fig. 1k–m). Picrosirius red staining combined with polarized light microscopy (PSR–PLM) suggested that in the vehicle-treated group, collagen fibers within the healing region exhibited strong birefringence and a highly aligned orientation, indicative of organized fibrillar architecture. In contrast, the DT-treated group displayed markedly reduced birefringence intensity and disorganized fiber orientation (Fig. 1k). qRT–PCR analysis demonstrated that injured tendons exhibited higher expression levels of *Tnc* and *Tnmd* at 14 Dpi (Fig. 1n, o). In contrast, the DT-treated group resulted in a marked reduction in the expression of these tendon-specific markers, indicating impaired tendon maturation. Notably, biomechanical testing demonstrated a significant decrease in ultimate tensile strength and a reduction in stiffness in DT-treated group tendons relative to controls (Fig. 1p, q). Collectively, these data indicate that p16^INK4a+^ cells accumulate during tendon repair and that their depletion compromises tendon collagen organization and mechanical recovery.

### p16^INK4a+^ cells acquire a pro-regenerative ECM secretory phenotype after tendon injury

**Fig. 2:**
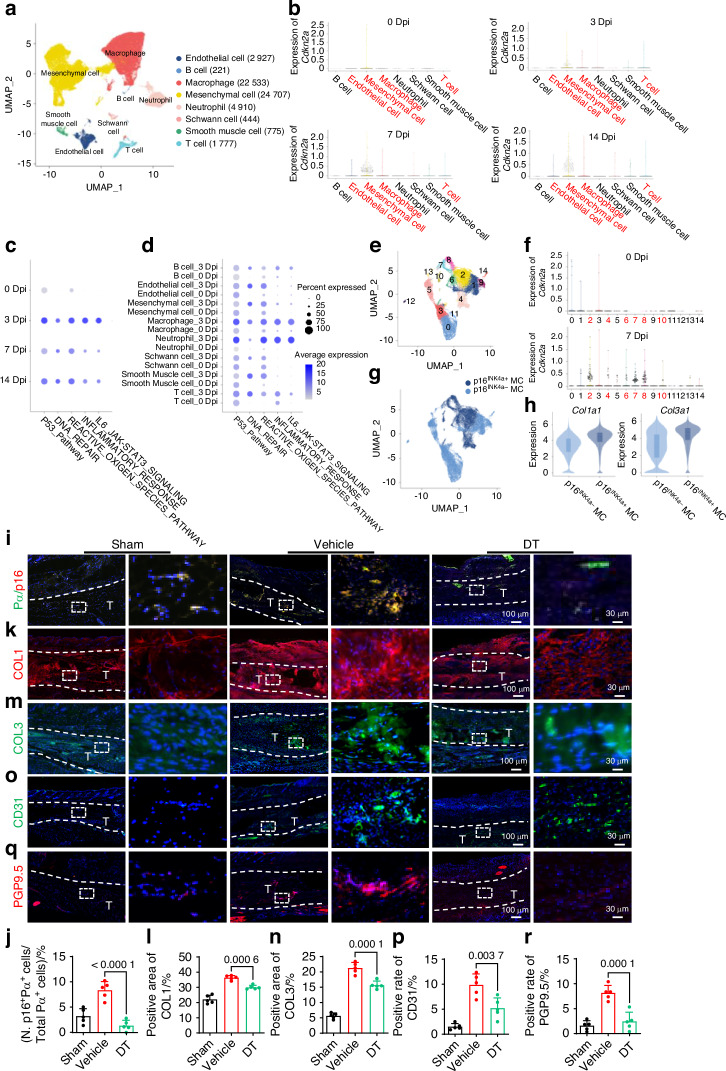
p16^INK4a+^ cells acquire a pro-regenerative ECM secretory phenotype after tendon injury. **a**–**h** Bioinformatic analysis of a previously published scRNA-seq dataset (PRJNA976191) of cells isolated from injured mouse tendons at multiple post-injury time points. **a** Uniform manifold approximation and projection (UMAP) visualization showing the distribution of major cell populations. **b** Feature plots showing the expression of *Cdkn2a* across all identified cell populations. **c** Pathway enrichment analysis (MSigDB) showing activation of related biological pathways associated with tendon injury at different time points after tendon injury. **d** Pathway enrichment analysis between uninjured tendons (0 Dpi) and injured tendons at 3 Dpi of eight cell populations. **e** UMAP visualization showing fourteen transcriptionally distinct mesenchymal cell subclusters. **f** Feature plots showing *Cdkn2a* expression levels across mesenchymal cell subclusters. **g** UMAP visualization showing the distribution of p16^INK4a+^ and p16^INK4a−^ mesenchymal cells. **h** Expression levels of extracellular matrix–related genes *Col1a1* and *Col3a1* in p16^INK4a+^ mesenchymal cells compared with p16^INK4a^^−^ mesenchymal cells. **i**–**r** Eight-week-old male *p16-iDTR* mice were subjected to a central puncture injury in the middle of the right Achilles tendon and treated with vehicle or diphtheria toxin (DT) to selectively ablate p16^INK4a+^ cells. Mice were sacrificed at 14 days post-injury. **i**, **j** Representative co-immunofluorescence images showing p16^INK4a^ (red) and the mesenchymal cell marker PDGFRα (green) in injured tendon sections are shown in (**i**) with quantification of p16^INK4a+^PDGFRα^+^ cells per mm² of tendon tissue in (**j**). Boxed regions are shown at higher magnification in the corresponding right panels. **k**–**r** Representative immunofluorescence images showing COL1 (**k**) COL3 (**m**) CD31^+^ blood vessels (**o**) and PGP9.5^+^ nerve fibers (**q**) within the healing region, with corresponding quantifications shown in (**l**) (**n**) (**p**) and (**r**) respectively. Boxed regions are shown at higher magnification in the corresponding right panels. T tendon area. *n* = 5 mice per group. Data are presented as mean ± SD. Statistical significance was determined by one-way ANOVA

To characterize the cellular identity of p16^INK4a+^ cells during tendon repair, we utilized a pre-established single-cell RNA sequencing (scRNA-seq) database on cells isolated from the injured mice tendons of multiple post-injury time points (PRJNA976191).^23^ Unbiased clustering analysis identified eight major cell populations (Fig. 2a), including endothelial cell, B lymphocyte, macrophage, mesenchymal cell, neutrophil, Schwann cell, smooth muscle cell, and T lymphocyte based on marker gene expression (Fig. S3A). Tendon injury was accompanied by infiltration of macrophages and neutrophils at 3 Dpi and massive activation of mesenchymal cells at 7 Dpi (Fig. S3B). Notably, senescence-associated markers were induced in both mesenchymal and immune compartments following tendon injury, but with distinct temporal dynamics. Specifically, *Cdkn1a* (*p21*) expression was broadly induced across multiple cell types, including mesenchymal cells, macrophages, neutrophils, endothelial cells, and T lymphocytes (Fig. S4A–D). In contrast, *Cdkn2a (p16)* expression was predominantly enriched in mesenchymal cells and became most prominent at 14 Dpi, indicating a dominant p16^INK4a+^ mesenchymal population after the initiation of the reparative phase of tendon repair (Fig. 2b). A detailed percentage of p16^INK4a+^ cells across different cell populations is provided in Table S1. Pathway enrichment analysis confirmed that early tendon injury at 3 Dpi was linked with increased enrichment intensity of p53 pathway, DNA repair, increased ROS&inflammation and activated IL-6–JAK–STAT3 pathway activity across all populations in general (Fig. 2c). GO pathway enrichment analysis further confirmed that the differentially expressed protein-coding genes (DEGs) were molecularly enriched in inflammatory response at 3 Dpi, collagen-containing extracellular matrix, angiogenesis at 7 Dpi and activation of p53 pathway at 14 Dpi (Fig. S5A–C). We further conducted signaling pathway enrichment analyses on all populations between 0 Dpi and 3 Dpi. Notably, mesenchymal cells, macrophages and neutrophils exhibited pronounced activation of the above pathways, highlighting their role as the important populations orchestrating the early reparative response within the injured tendon (Fig. 2d). Interestingly, activation of the mesenchymal population was accompanied by an increase in mtDNA copy number, indicating altered metabolic demands and a potential enhancement of mitochondrial biogenesis to support mesenchymal cells activation (Fig. S6A). To further examine the long-term trajectory of p16^INK4a+^ cells, we analyzed an independent publicly available human tendon injury dataset (PRJNA975881) comparing early (3 days) and late (12 weeks) post-injury time points (Fig. S7A). PDGFRα was identified as a marker for mesenchymal cells (Fig. S7B). In this dataset, p16^INK4a+^ mesenchymal cells showed a modest reduction at 12 weeks post-injury compared with 3 Dpi or 10 Dpi, while remaining detectable in mesenchymal cells (Fig. S7C). Given that mesenchymal cells were identified as the major p16^INK4a+^ population, we assessed the transcriptional features of these cells by performing DEGs analysis comparing mesenchymal cells from 3, 7, and 14 Dpi with those from uninjured tendons (0 Dpi). Heatmap visualization revealed that mesenchymal cells at 7 and 14 Dpi displayed a strong upregulation of ECM-related genes, especially *Col3a1*, relative to baseline (Fig. S8A–C). suggesting that mesenchymal population adopt a reparative, matrix-productive phenotype during tendon repair. To explore if p16^INK4a^ expression was associated with collagen synthesis, we next conducted subcluster analysis to further delineate the expression level of p16^INK4a^ and functional states of p16^INK4a+^ mesenchymal cells during tendon repair. A total of fourteen distinct mesenchymal cell subclusters were identified (Fig. 2e), which could be broadly categorized into p16^INK4a+^ and p16^INK4a^^−^ mesenchymal cell populations (p16^INK4a+^ MC vs p16^INK4a^^−^ MC) based on the expression of p16^INK4a^ (Fig. 2f, g). We compared the expression of extracellular matrix–related genes between p16^INK4a+^ and p16^INK4a^^−^ mesenchymal cell populations. Notably, p16^INK4a+^ mesenchymal cells exhibited markedly higher expression levels of key ECM genes *Col1a1* and *Col3a1*, indicating that p16^INK4a+^ mesenchymal cells actively participate in collagen synthesis and matrix remodeling rather than representing a purely degenerative state (Fig. 2h). To further determine the functional role of these p16^INK4a+^ cells in tendon repair, we examined tendons from *p16-iDTR* mice. Immunofluorescence staining of Achilles tendon sections validated our findings: p16^INK4a+^ cells were mostly localized within the injured tissue and displayed extensive co-localization with mesenchymal cell marker PDGFRα (Fig. 2i, j). Immunofluorescence of ECM components revealed a pronounced reduction in COL1 (Fig. 2k, l) and COL3 expression (Fig. 2m, n) in injured tendons of DT-treated mice compared with vehicle-treated mice, suggesting that ablation of p16^INK4a+^ cells affected collagen composition during tendon repair. Recent publications highlighted the importance of angiogenesis and innervation during tissue repair.^24–26^ Interestingly, the density of CD31^+^ vasculature (Fig. 2o, p) and PGP9.5^+^ nerve fibers (Fig. 2q, r) in the healing zone was also markedly reduced in DT-treated mice. To explore the mechanism linking p16^INK4a+^ cells to angiogenesis and neo-innervation during tendon repair, we examined the expression of angiogenic and neurotrophic factors. scRNA-seq analysis revealed that DEGs were enriched in the angiogenesis pathway and the neurogenesis pathway in p16^INK4a^^−^ mesenchymal cell (Fig. S9A). Further analysis proved that p16^INK4a+^ mesenchymal cells express significantly higher levels of pro-angiogenic factors, including *Vegfa* and *Pdgfa*, as well as neurotrophic factors *Ngf* and *Bdnf*, compared with p16^INK4a^^−^ mesenchymal cells (Fig. S9B). Collectively, these findings demonstrate that p16^INK4a+^ mesenchymal cells display higher ECM synthetic activity and secrete paracrine cues that support matrix organization, neovascularization, and neural ingrowth. Ablation of these cells disrupts the reparative microenvironment and compromises functional tendon regeneration.

### Epigenetic activation of JMJD3 in p16^INK4a+^ cells after tendon injury

**Fig. 3:**
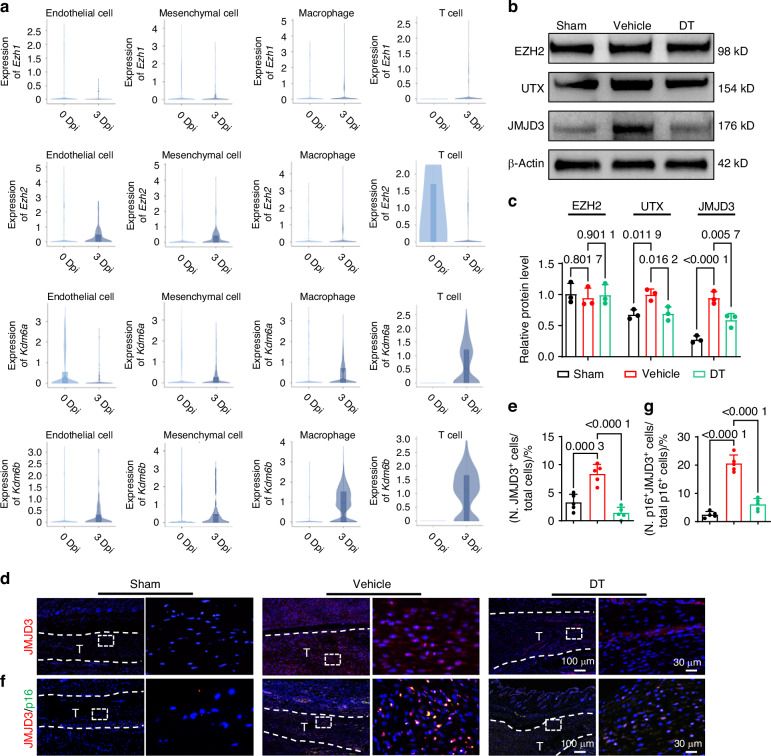
Epigenetic activation of JMJD3 in p16^INK4a+^ cells after tendon injury. **a** Violin plots showing the expression levels of histone methylation regulators *Ezh1*, *Ezh2*, *Utx* (*Kdm6a*), and *Jmjd3* (*Kdm6b*) across four major p16^INK4a+^ cell populations. **b**, **c** Eight-week-old male *p16-iDTR* mice were subjected to a central puncture injury in the middle of the right Achilles tendon and treated with vehicle or diphtheria toxin (DT). Representative Western blot showing JMJD3 expression in injured tendons is shown in (**b**) with quantification shown in (**c**). **d**, **e** Representative immunofluorescence images showing JMJD3 expression (red) in injured tendon sections in (**d**) with quantification of JMJD3^+^ cells per mm² of tendon tissue in (**e**). Boxed regions are shown at higher magnification in the corresponding right panels. **f**, **g** Representative immunofluorescence images showing co-localization of JMJD3 (red) and p16^INK4a^ (green) in healing tendon sections in (**f**) with quantification of JMJD3^+^p16^INK4a+^ cells per mm² of tendon tissue in (**g**). Boxed regions are shown at higher magnification in the corresponding right panels. T tendon area. *n* = 5 mice per group. Data are presented as mean ± SD. Statistical significance was determined by one-way ANOVA

Epigenetic regulation has emerged as a fundamental mechanism controlling both the onset of cell-cycle-related gene expression and the activation of tissue repair programs.^27,28^ To delineate the epigenetic changes associated with p16^INK4a+^ cells during tendon healing, we analyzed the expression of key histone methylation regulators in scRNA-seq dataset on injured mouse tendons (Fig. 3a). The analysis focused on previous mentioned four major cell populations that express p16^INK4a^—mesenchymal cells, macrophages, endothelial cells, and T cells—which collectively represent most p16^INK4a+^ cell types during tendon repair. We first examined the expression of histone methyltransferases *Ezh1* and *Ezh2*, the catalytic subunits of Polycomb Repressive Complex 2 (PRC2) responsible for H3K27 trimethylation.^29^ *Ezh1* showed minimal or nearly undetectable expression across all four clusters. In contrast, *Ezh2* was significantly upregulated in mesenchymal cells and endothelial cells after tendon injury, while exhibiting very low expression in macrophages and T cells after injury. Next, we analyzed the histone demethylase *Utx* (also known as *Kdm6a*), which catalyzes the removal of H3K27me3 marks and functionally antagonizes *Ezh2*. *Utx* displayed divergent, cell-type–dependent regulation: its expression was increased in mesenchymal cells, macrophages, and T lymphocytes, but was reduced in endothelial cells. Finally, the demethylase *Jmjd3* (also known as *Kdm6b*) was found to be consistently and robustly upregulated across all p16^INK4a+^ populations, with the most pronounced induction in immune cells (macrophages and T cells), followed by moderate elevation in mesenchymal cells and endothelial cells. Previous findings have identified JMJD3 as a key regulator of ECM component hyaluronic acid (HA) during muscle repair.^30^ The widespread increase of JMJD3 expression indicates that chromatin relaxation via active demethylation may be a shared feature of p16^INK4a+^ cells in the healing tendon. To validate whether these epigenetic changes were associated with p16^INK4a^ induction in the regenerating microenvironment, we used *p16-iDTR* mice, and JMJD3 expression was examined after p16^INK4a+^ cell ablation. The level of JMJD3 was elevated after tendon injury but was markedly diminished upon DT administration (Fig. 3b, c), while the levels of EZH2 and UTX were less affected, supporting the activation of JMJD3 in p16^INK4a+^ cells. Immunofluorescence staining corroborated these findings: JMJD3 was highly expressed in the tendon healing tissue (Fig. 3d, e). However, in DT-treated mice, both the intensity and abundance of JMJD3-expressing nuclei decreased dramatically. Consistently, JMJD3-expressing p16^INK4a+^ cells were also markedly decreased in the DT-treated group (Fig. 3f, g). Thus, tendon injury-induced p16^INK4a+^ cells in the lesion area, with activation of JMJD3 as one of the main characteristics.

### JMJD3-mediated H3K27me3 demethylation activates ECM synthesis in p16^INK4a+^ cells

**Fig. 4:**
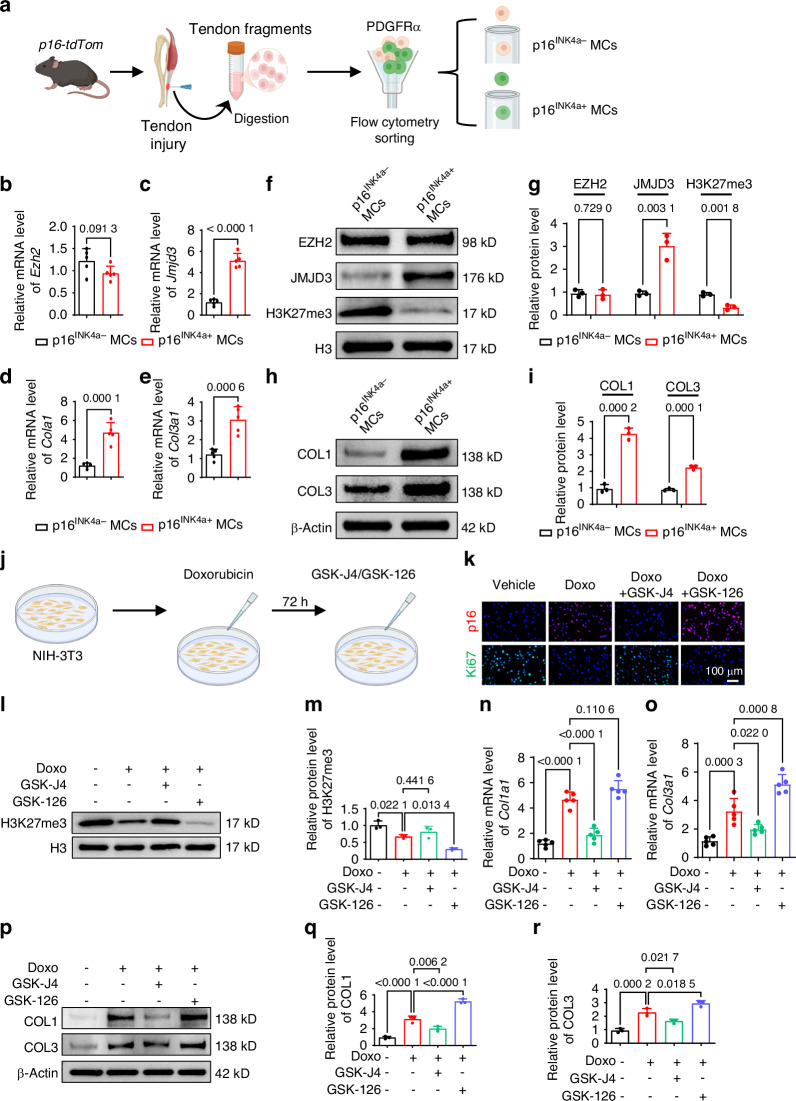
JMJD3-mediated H3K27me3 demethylation activates ECM synthesis in p16^INK4a+^ cells. **a** Schematic overview of the experimental workflow for isolating p16^INK4a+^ and p16^INK4a−^ mesenchymal cells. tdTom^+^ and tdTom⁻ mesenchymal cells were sorted from injured tendons of *p16-tdTom* reporter mice using PDGFRα as a mesenchymal cell marker. Quantitative RT–PCR analysis showing the expression levels of *Ezh2* (**b**) and *Jmjd3* (**c**) in p16^INK4a+^ and p16^INK4a−^ mesenchymal cells. Quantitative RT–qPCR analysis showing the expression levels of *Col1a1* (**d**) and *Col3a1* (**e**) in p16^INK4a+^ and p16^INK4a−^ mesenchymal cells. Representative Western blot showing EZH2, JMJD3, and H3K27me3 protein levels in p16^INK4a+^ and p16^INK4a−^ mesenchymal cells in (**f**) with quantification shown in (**g**). Representative Western blot showing COL1 and COL3 protein levels in p16^INK4a+^ and p16^INK4a−^ mesenchymal cells in (**h**) with quantification shown in (**i**). **j**–**r** NIH-3T3 cells were treated with doxorubicin (250 nmol/L) in the presence or absence of GSK-J4 (10 μmol/L) or GSK-126 (5 μmol/L). **j** Schematic diagram showing the experimental design of the in vitro model. **k** Representative immunofluorescence images showing p16^INK4a^ and Ki67 expression in NIH-3T3 cells. Representative Western blot showing H3K27me3 protein levels in NIH-3T3 cells treated with GSK-J4 or GSK-126 (**l**) with quantification shown in (**m**). Quantitative RT–qPCR analysis showing the expression levels of *Col1a1* (**n**) and *Col3a1* (**o**) in NIH-3T3 cells treated with GSK-J4 or GSK-126. Representative Western blot showing COL1 and COL3 protein levels in NIH-3T3 cells treated with GSK-J4 or GSK-126 in (**p**) with quantification shown in (**q**) and (**r**). *n* = 5 mice per group for in vivo experiments and *n* = 3 for in vitro Western blot analyses. Data are presented as mean ± SD. Statistical significance was determined using unpaired two-tailed Student’s *t*-tests or one-way ANOVA, as appropriate

To determine whether JMJD3 is activated in p16^INK4a+^ mesenchymal cells in vivo, we isolated p16^INK4a+^ and p16^INK4a^^−^ mesenchymal cells (MCs) using PDGFRα as a mesenchymal cell marker from Achilles tendons of *p16-tdTom* reporter mice using FACS (Fig. 4a). qRT–PCR analysis revealed a slight downregulation of *Ezh2* and a marked upregulation of *Jmjd3* in p16^INK4a+^ mesenchymal cells compared with p16^INK4a−^ counterpart (Fig. 4b, c). In parallel, *Col1a1* and *Col3a1* mRNA levels were significantly elevated in the p16^INK4a+^ mesenchymal cell population (Fig. 4d, e). At the protein level, consistently, Western blot analysis revealed elevated JMJD3 protein level, accompanied by an overall decrease in H3K27me3 in p16^INK4a+^ mesenchymal cells compared with p16^INK4a−^ mesenchymal cells (Fig. 4f, g). The protein level of COL1 and COL3 was also markedly higher in p16^INK4a+^ mesenchymal cells than in p16^INK4a^^−^ mesenchymal cells (Fig. 4h, i). qRT–PCR analysis demonstrated that p16^INK4a+^ mesenchymal cells express higher expression levels of pro-angiogenic factors and neurotrophic factors compared with p16^INK4a^^−^ mesenchymal cells (Fig. S10A, B). These findings demonstrate that JMJD3 activation and H3K27me3 demethylation are closely associated with the enhanced collagen-synthetic and -remodeling activity of p16^INK4a+^ cells. To further confirm the causal relationship between JMJD3 activation and extracellular matrix gene expression, we established an in vitro model of a doxorubicin (Doxo)-induced p16^INK4a+^ mesenchymal cell state (Fig. 4j). Exposure of NIH-3T3 cell line to Doxo (250 nmol/L) induced elevated expression of p16, accompanied by reduced levels of Ki67 (Fig. 4k). Notably, H3K27me3 marks were markedly reduced at the protein level in Doxo-induced p16^INK4a+^ mesenchymal cells (Fig. 4l, m). To determine whether modulation of histone H3 methylation/demethylation enzymatic activity affects epigenetic state and collagen synthesis, we treated p16^INK4a+^ mesenchymal cells with the JMJD3 inhibitor GSK-J4 (10 μmol/L) or the EZH2 inhibitor GSK-126 (5 μmol/L) for 48 h. Western blotting confirmed that GSK-J4 restored H3K27me3 accumulation, while GSK-126 diminished the H3K27me3 mark (Fig. 4l, m). GSK-J4 treatment significantly suppressed the expression of *Col1a1* and *Col3a1*, whereas GSK-126 treatment enhanced their expression in Doxo-induced p16^INK4a+^ mesenchymal cells at both mRNA and protein levels (Fig. 4n–r). These results indicate that JMJD3-mediated demethylation of H3K27me3 is essential for the activation of collagen synthesis in p16^INK4a+^ mesenchymal cells.

### Loss of JMJD3 in p16^INK4a+^ cells impairs tendon repair

**Fig. 5:**
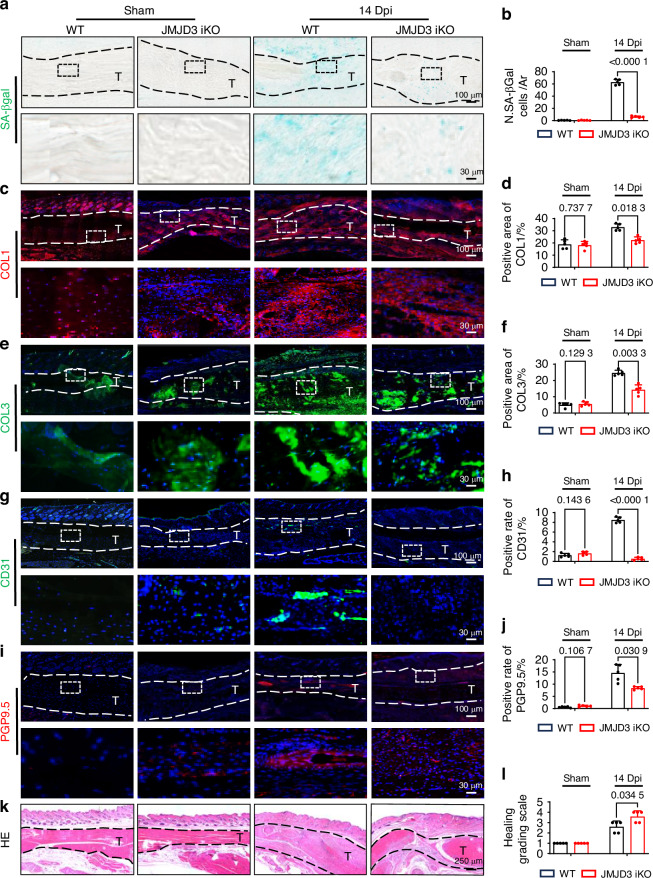
Loss of JMJD3 in p16^INK4a+^ cells impairs tendon repair. Eight-week-old male *p16-Cre*^*ERT2*^*::JMJD3*^*flox/flox*^ (*JMJD3 iKO*) and wild-type (*JMJD3*^*flox/flox*^) mice were subjected to a central puncture injury in the middle of the right Achilles tendon and sacrificed at 14 Dpi. Representative images of SA-βGal staining in tendon sections are shown in (**a**) with quantification of SA-βGal^+^ cells per mm² of tendon tissue area in (**b**). Boxed regions are shown at higher magnification in the corresponding lower panels. Representative immunofluorescence images showing COL1 expression in tendon sections in (**c**) with quantification of COL1-positive area per mm² of tendon tissue area in (**d**). Boxed regions are shown at higher magnification in the corresponding lower panels. Representative immunofluorescence images showing COL3 expression in tendon sections in (**e**) with quantification of COL3-positive area per mm² of tendon tissue area in (**f**). Boxed regions are shown at higher magnification in the corresponding lower panels. Representative immunofluorescence images showing CD31 expression in tendon sections in (**g**) with quantification of CD31-positive area per mm² of tendon tissue area in (**h**). Boxed regions are shown at higher magnification in the corresponding lower panels. Representative immunofluorescence images showing PGP9.5 expression in tendon sections in (**i**) with quantification of PGP9.5-positive area per mm² of tendon tissue area shown in (**j**). Boxed regions are shown at higher magnification in the corresponding lower panels. Representative hematoxylin and eosin (H&E)–stained sections of injured tendons in (**k**) with quantification of histological parameters shown in (**l**). T tendon area. *n* = 5 mice per group. Data are presented as mean ± SD. Statistical significance was determined using multiple unpaired two-tailed Student’s *t*-tests

To determine whether JMJD3-driven demethylation of H3K27me3 is essential for the pro-regenerative function of p16^INK4a+^ cells during tendon healing, we generated *p16-Cre*^*ERT2*^*::JMJD3*^*flox/flox*^ mice (*JMJD3 iKO* mice), in which JMJD3 is inducibly deleted in p16^INK4a+^ cells. SA-β-gal staining showed a significant reduction in p16^INK4a+^ cells within the injured *JMJD3 iKO* tendons compared with wild-type (WT) controls at 14 Dpi (Fig. 5a, b). Immunofluorescence staining revealed a pronounced reduction in COL1 and COL3 expression in injured tendons of *JMJD3 iKO* mice compared with WT mice, suggesting disrupted collagen deposition (Fig. 5c–f). Furthermore, the density of CD31^+^ vasculature and PGP9.5^+^ nerve fibers in the healing zone was markedly reduced in *JMJD3 iKO* mice (Fig. 5g–j), suggesting that loss of JMJD3 in p16^INK4a+^ cells impairs angiogenesis and innervation during tendon repair. H&E staining suggested *JMJD3 iKO* tendons exhibited disrupted collagen fiber alignment, reduced cross-sectional area, and decreased cellular density in the healing interface (Fig. 5k, l). Collectively, these results indicate that the elevation of H3K27me3 levels in p16^INK4a+^ cells, achieved through JMJD3 ablation, effectively reduced collagen deposition and ECM remodeling in this region and impaired tendon regeneration.

### Pharmacological modulation of H3K27 methylation affects tendon repair

**Fig. 6:**
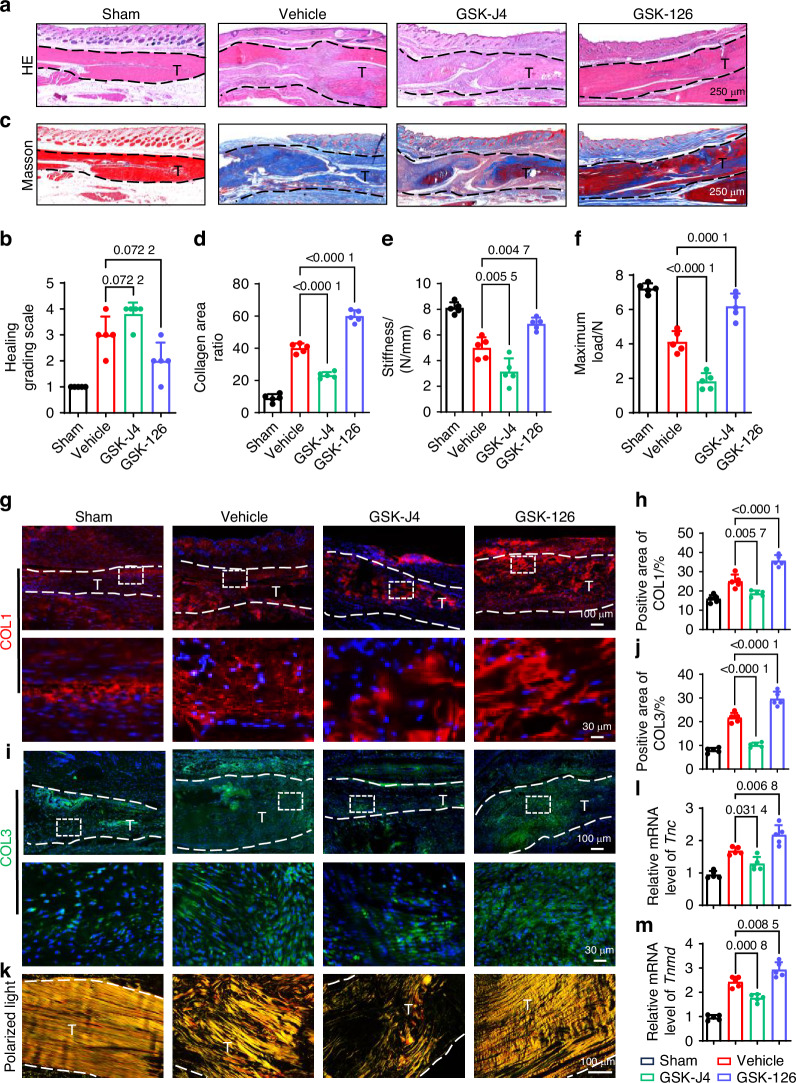
Pharmacological modulation of H3K27 methylation affects tendon repair. Eight-week-old male C57BL/6J mice were subjected to Achilles tendon puncture injury and treated with vehicle, the JMJD3 inhibitor GSK-J4 (2 mg/kg), or the EZH2 inhibitor GSK-126 (10 mg/kg), and sacrificed at 14 Dpi. Representative histological images of injured tendons stained with hematoxylin and eosin (H&E) (**a**) and Masson’s trichrome (**c**) with quantification of histological parameters shown in (**b**) and (**d**) respectively. Biomechanical evaluation of healing tendons showing tendon stiffness (**e**) and maximum load (**f**). Representative immunofluorescence images showing COL1 expression (red) in (**g**) and COL3 expression (green) in (**i**) with quantification of COL1-positive area and COL3-positive area per mm² of tendon tissue area shown in (**h**) and (**j**) respectively. Boxed regions are shown at higher magnification in the corresponding lower panels. **k** Representative images of tendon sections stained with picrosirius red and visualized under polarized light microscopy. Quantitative RT–qPCR analysis showing the relative mRNA expression levels of tendon-related genes *Tnc* (**l**) and *Tnmd* (**m**). T, tendon area. *n* = 5 mice per group. Data are presented as mean ± SD. Statistical significance was determined using one-way ANOVA

To validate the causal role of H3K27 methylation dynamics in tendon regeneration, we pharmacologically manipulated both the demethylase (JMJD3) and methyltransferase (EZH2) arms of this axis using their inhibitors GSK-J4 or GSK-126, respectively.^31,32^ Eight-week-old male WT mice were subjected to Achilles tendon puncture and treated with vehicle, GSK-J4 (2 mg/kg), or GSK-126 (10 mg/kg) during the early healing phase (14 Dpi). As described above, GSK-J4 administration led to a reduction in ECM synthesis and impaired tendon healing (Fig. 6a–d). In contrast, inhibition of EZH2 with GSK-126 produced better phenotypic outcomes. At the tissue level, Masson’s trichrome staining demonstrated more organized collagen alignment and denser interfascicular matrix in GSK-126–treated tendons compared with vehicle or GSK-J4 group (Fig. 6c, d). Functionally, biomechanical testing indicated that GSK-126 treatment restored the ultimate tensile strength and stiffness of repaired tendons compared with GSK-J4–treated tendons (Fig. 6e, f). Immunofluorescence staining revealed that GSK-126 treatment increased ECM protein COL1 and COL3 (Fig. 6g–j). PSR–PLM results suggested that JMJD3 inhibition significantly decreased fiber orientation coherence (Fig. 6k). qRT–PCR analysis revealed that JMJD3 inhibition by GSK-J4 significantly reduced the expression of *Tnc* and *Tnmd* in injured tendons compared with vehicle-treated controls. In contrast, EZH2 inhibition by GSK-126 exerted an opposing effect, resulting in partially restored expression of these tendon markers relative to the vehicle group (Fig. 6l, m). Together, these pharmacological effects underscore the functional necessity of dynamic H3K27 methylation in balancing regenerative matrix synthesis and ultimate tendon repair outcome.

## Discussion

**Fig. 7:**
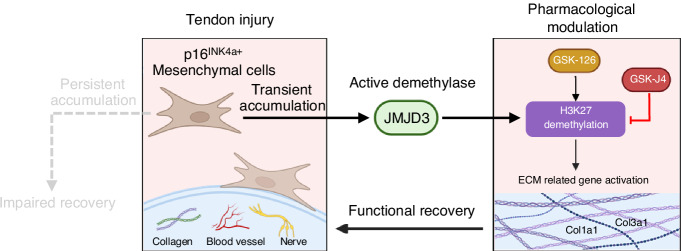
Schematic model of the JMJD3–H3K27me3 axis in tendon regeneration. Following tendon injury, transiently accumulated p16^INK4a+^ mesenchymal cells upregulate JMJD3, leading to demethylation of H3K27me3 and activation of ECM-related genes. This promotes collagen organization, angiogenesis, and nerve ingrowth, thereby facilitating tendon repair. Loss or pharmacological inhibition of JMJD3 (GSK-J4) restores H3K27me3 levels, suppresses ECM synthesis, and impairs tendon healing. In contrast, inhibition of EZH2 (GSK-126) enhances collagen deposition and improves functional recovery of injured tendons

Our study identifies a previously unappreciated role of p16^INK4a^−positive cells in functional tendon regeneration through an epigenetic mechanism governed by JMJD3-mediated H3K27me3 demethylation (Fig. 7). Using p16 reporter and inducible ablation mouse models, we demonstrate that p16^INK4a+^ cells accumulate during tendon healing, and their depletion markedly impairs collagen organization, vascularization, innervation, and final mechanical recovery. Single-cell transcriptomics reveal that p16^INK4a+^ mesenchymal cells are one of the sources of ECM components, exhibiting a pro-regenerative transcriptional program characterized by enhanced collagen synthesis and matrix remodeling. Mechanistically, we show that JMJD3 is robustly activated in p16^INK4a+^ mesenchymal cells, leading to the removal of repressive H3K27me3 marks and transcriptional activation of ECM-related genes. Genetic or pharmacological inhibition of JMJD3 suppresses collagen synthesis and compromises tendon repair, whereas inhibition of the opposing enzyme EZH2 enhances regenerative outcomes.

Although p16^INK4a+^ cells have been widely associated with aging and the fibrosis process, accumulating evidence suggests that transient accumulation of p16^INK4a+^ cells with senescent-associated characteristics can play beneficial roles in tissue regeneration by coordinating immune and stromal responses.^11,12^ In line with previous reports on skin and tissue repair, we observed the presence of p16^INK4a+^ cells that peaked around day 14 Dpi, coinciding with the initiation of proliferative and matrix-deposition phases of tendon healing. Our long-term analyses in human datasets indicate that resolution of injury-associated p16^INK4a^ upregulation is gradual rather than abrupt. In human tendons, p16^INK4a^ expression exhibited a modest decline at late healing stages, while remaining detectable in a subset of stromal cells. This pattern suggests that transient p16^INK4a^ expression during tendon repair should be defined by progressive attenuation rather than complete elimination of p16^INK4a+^ cells. Such gradual resolution is consistent with the slow remodeling kinetics of tendon tissue and supports a model in which injury-activated p16^INK4a+^ mesenchymal cells transition toward a post-senescent state. We found that functional depletion of p16^INK4a+^ cells disrupted collagen fiber alignment and diminished biomechanical strength, underscoring their indispensable role in early matrix organization. These findings indicate that the transient accumulation of p16^INK4a+^ cells in healing tendon is not merely a byproduct of cellular stress but a temporally restricted, adaptive program that supports tissue repair. Such dualistic behavior of the p16^INK4a^-associated senescence-like program—detrimental when chronic yet beneficial when transient—highlights the importance of context-dependent regulation in tissue repair. It is worth noting that we do not propose p16^INK4a^ expression as an inherently beneficial cellular state. Instead, our data supports that a temporally restricted, acute senescence-associated response may contribute positively to tendon repair, whereas a persistent or unresolved senescence-associated state is likely to be deleterious, consistent with extensive literature in aging, fibrosis, and degenerative diseases.^33,34^

Single-cell RNA sequencing allowed us to resolve the cellular heterogeneity of p16^INK4a+^ cells within the healing tendon. Our analysis demonstrated that, although both immune and mesenchymal populations exhibit some of the senescence-associated markers following injury, these responses are molecularly and temporally distinct. Immune cells predominantly display a transient p21^CIP1^ expression during the early inflammatory phase, whereas mesenchymal cells constitute the dominant p16^INK4a+^ population during the matrix-deposition and remodeling phase. Importantly, p16^INK4a+^ mesenchymal cells displayed high expression of *Col1a1* and *Col3a1* and activation of DNA repair and IL-6–JAK–STAT3 pathways, suggesting that these cells remain metabolically active and participate in matrix remodeling rather than undergoing terminal deterioration. In vivo, p16^INK4a+^ cells colocalized with PDGFRα^+^ mesenchymal cells and produced more collagen-rich ECM than p16^INK4a^^−^ mesenchymal cells, while their depletion led to disorganized collagen architecture and reduced angiogenesis and innervation. This aligns with recent evidence that p16^INK4a+^ cells can function as transient “matrix organizers,” secreting both structural and trophic factors to support vascular and neural integration during tissue repair.

A key mechanistic insight from our work is the discovery that JMJD3, a H3K27me3 demethylase, acts as a central epigenetic effector in p16^INK4a+^ mesenchymal cells during tendon repair. Our scRNA-seq analysis revealed robust JMJD3 upregulation across p16^INK4a+^ cells, whereas EZH2—the methyltransferase responsible for H3K27me3 deposition—was slightly downregulated. This reciprocal regulation suggests a global chromatin-opening state in p16^INK4a+^ cells. Consistently, isolated p16^INK4a+^ mesenchymal cells exhibited higher JMJD3 expression, reduced H3K27me3 levels, and enhanced *Col1a1* and *Col3a1* transcription compared with p16^INK4a^^−^ mesenchymal cells. Pharmacological inhibition of JMJD3 by GSK-J4 restored H3K27me3 accumulation and suppressed collagen gene expression, whereas EZH2 inhibition by GSK-126 promoted collagen synthesis and improved mechanical strength in vivo. Together, these results demonstrate that JMJD3-mediated demethylation of H3K27me3 is required for the activation of regenerative ECM gene networks in p16^INK4a+^ mesenchymal cells. This mechanism resembles JMJD3’s role in other regenerative contexts, such as macrophage polarization, skeletal muscle regeneration, and osteogenic differentiation, where it facilitates lineage-specific transcriptional activation by erasing repressive histone marks.^30,35,36^ JMJD3 not only licenses transcription of ECM genes but may also shape the paracrine signaling landscape that regulates immune and endothelial responses. Previous studies have shown that JMJD3 can activate key regenerative transcription factors, including IRF4, HOXA genes, and Runx2, by demethylating their promoters.^37–39^ In tendon mesenchymal cells, similar chromatin remodeling may enable transcription of collagen and matrix assembly genes, supporting tissue restoration. Conversely, loss of JMJD3, either genetically or pharmacologically, results in elevated H3K27me3 levels and repressive chromatin, mimicking a chronically fibrotic state characterized by dysregulated ECM deposition and impaired angiogenesis. Thus, fine-tuning the balance between JMJD3 and EZH2 activity is crucial for maintaining a transient, pro-repair p16^INK4a+^ cell-associated program without tipping into pathological fibrosis.

The identification of an epigenetic signaling linked with reparative cells with senescent characteristics provides a possible translational perspective for tendon biology. Specifically, short-term EZH2 inhibition or transient JMJD3 activation during the early repair phase may amplify the beneficial effects of p16^INK4a+^ mesenchymal cells while avoiding persistent accumulation of p16^INK4a+^ cells or chronic senescence-associated cell states. However, temporal precision will be critical: sustained JMJD3 activation could lead to prolonged SASP and tissue degeneration, whereas premature inhibition may blunt early regenerative cues. Also, systemic administration of epigenetic inhibitors such as GSK-J4 and GSK-126 is inherently non–cell-type specific. In particular, GSK-J4 has been reported to exert immunomodulatory and off-target effects in immune cells and other mesenchymal compartments.^40,41^ Future studies employing time-resolved epigenomic profiling and lineage-specific manipulation of JMJD3 activity will be necessary to dissect these dynamics. Moreover, although our genetic and pharmacological data support a functional association between JMJD3 activity, p16^INK4a+^ mesenchymal cells, and tendon repair outcomes, future studies employing chromatin profiling approaches will be required to establish causal epigenetic relationships at the gene level.

In summary, our findings redefine the role of p16^INK4a+^ cells in tendon healing, revealing that transient accumulation of p16^INK4a+^ cells promotes regeneration through JMJD3-mediated epigenetic reprogramming of ECM genes. Given that p16^INK4a+^ mediated senescence-associated features and chromatin remodeling are conserved processes across connective tissues, the JMJD3–H3K27me3 axis may have broader implications for wound healing, muscle regeneration, and fibrosis resolution. Targeting this pathway offers a promising avenue for designing time-specific, epigenetically informed therapies to enhance regeneration and prevent fibrotic healing.

## Materials And Methods

### Experimental animals and treatments

*C57BL/6J* (Strain No. N000013), *Cdkn2a-Cre*^*ERT2*^ (Strain No. NM-KI-18039), and *Kdm6b*^*flox/flox*^ (Strain No. NM-CKO-200113) mice were obtained from GemPharmatech (Nanjing, China) and Shanghai Model Organisms Center (Shanghai, China). *Rosa26-iDTR* mice were purchased from The Jackson Laboratory (Bar Harbor, ME, USA). For conditional ablation of p16^INK4a+^ cells, *Cdkn2a-Cre*^*ERT2*^ mice were crossed with *Rosa26-iDTR* mice to generate *Cdkn2a-Cre*^*ERT2*^*::iDTR (p16-iDTR)* mice. To assess the role of JMJD3-mediated H3K27me3 demethylation in tendon repair, *Cdkn2a-Cre*^*ERT2*^ mice were bred with *Jmjd3*^*flox/flox*^ mice to obtain *Cdkn2a-Cre*^*ERT2*^*::Jmjd3*^*flox/flox*^ (*JMJD3 iKO*) mice. Cre activity was induced by intraperitoneal injection of tamoxifen (100 mg/kg, Sigma-Aldrich, T5648) once daily for five consecutive days.^42^ Diphtheria toxin (DT) (Sigma-Aldrich, D0564) was dissolved in PBS and was injected (10 ng/g body weight per time) three times per week.^43^ Genotypes were verified by PCR using genomic DNA from tail or toe biopsies following the manufacturer’s instructions. All animals were housed under specific pathogen-free (SPF) conditions with a 12-h light/dark cycle and free access to food and water. All procedures were approved by the Institutional Animal Care and Use Committee of Shanghai Jiao Tong University (A2024434-002).

For the Achilles tendon injury model, 8-week-old male mice were anesthetized with isoflurane, and a single puncture was made at the midportion of the right Achilles tendon using a 21-gauge needle. The skin incision was closed with 6-0 nylon sutures, and mice were allowed free cage activity postoperatively. Tendons were collected at 0, 7, and 14 days after injury and processed for histological or molecular analysis.

For pharmacological studies, mice received intraperitoneal injections of GSK-J4 (2 mg/kg; MedChemExpress, HY-15648B) or GSK-126 (10 mg/kg; MedChemExpress, HY-13470) daily for 14 days after injury. Doses were selected based on reported in vivo usage ranges with attention to avoid high-dose systemic effects. The GSK-J4 dose (2 mg/kg, i.p.) was selected within the low mg/kg range reported for systemic in vivo inhibition of H3K27 demethylase activity (e.g., 0.5 mg/kg daily and 1–3 mg/kg in inflammatory models).^44,45^ The GSK-126 dose (10 mg/kg, i.p.) was selected based on prior in vivo studies reporting effective EZH2 pathway modulation at 10 mg/kg in murine models.^46,47^ Both compounds are reversible epigenetic inhibitors and were therefore administered daily for 14 days post-injury to maintain sustained pathway modulation. Vehicle-treated mice (5% DMSO) served as controls.

### Biomechanical testing

Mechanical properties of tendons were evaluated at 14 Dpi using a dynamic mechanical analyzer (Q800, TA Instruments).^48^ Following dissection, tendon specimens were immediately wrapped in phosphate-buffered saline (PBS)-soaked gauze and stored at −80 °C until testing. Prior to mechanical testing, specimens were thawed at room temperature and kept hydrated in PBS to preserve tissue mechanical integrity. The tendon was secured in a clamp, and the proximal tendon end was gripped using sandpaper-lined clamps to prevent slippage. The initial distance between clamps was measured prior to loading. Tendons were loaded with uniaxial tension at a constant displacement rate of 1 mm/min until failure. Force and displacement were continuously recorded throughout the test.

### Immunofluorescence staining of Achilles tendon sections

For histological evaluation, the Achilles tendon was dissected, fixed in 4% paraformaldehyde overnight at 4 °C, dehydrated, and paraffin-embedded until sectioning. Longitudinal sections (4 µm) were prepared and subjected to hematoxylin–eosin (H&E) and Masson’s trichrome staining following standard protocols. For frozen sections, freshly fixed tendons were washed with PBS, dehydrated in 20% sucrose containing 2% polyvinylpyrrolidone for 24 h at 4 °C, embedded in O.C.T. compound (Sakura Finetek), and sectioned longitudinally at 20 µm thickness. Cellular senescence was visualized using a Senescence β-Galactosidase Staining Kit (Cell Signaling Technology) according to the manufacturer’s instructions.

For immunofluorescence staining, cryosections were permeabilized with 0.2% Triton X-100 for 10 min, blocked in 5% bovine serum albumin (BSA), and incubated overnight at 4 °C with the following primary antibodies: JMJD3 (Abcam, ab38113), PDGFRα (Cell Signaling Technology, 3174), γH2AX (Abcam, ab81299), p16 (Abcam, ab26696), COL1A1 (Cell Signaling Technology, 72026), COL3A1 (Proteintech, 22734-1-AP), CD31 (Abcam, ab7388), and PGP9.5 (Abcam, ab108986). After washing, sections were incubated for 1 h at room temperature with Alexa Fluor® 488- or 594-conjugated secondary antibodies (Jackson ImmunoResearch). Nuclei were counterstained with DAPI (Vector Laboratories, H-1200) and mounted with antifade medium. Confocal images were captured using a Zeiss LSM 780 microscope (Carl Zeiss, Germany), and fluorescence intensity was quantified using ImageJ software (NIH).

### Picrosirius red staining and polarized light microscopy (PSR–PLM)

Paraffin-embedded Achilles tendon sections were deparaffinized, rehydrated through graded ethanol, and stained with 0.1% picrosirius red solution (Direct Red 80 in saturated aqueous picric acid) for 60 min. Sections were then rinsed in acidified water, dehydrated, cleared, and mounted with a non-aqueous mounting medium. Stained sections were imaged under polarized light using a polarized light microscope equipped with crossed polarizers (Leica DM6 B POL).

### Cell culture, drug treatments, and immunofluorescence staining

NIH-3T3 cells (ATCC, USA) were maintained in Dulbecco’s modified Eagle’s medium (DMEM, high glucose) supplemented with 10% fetal bovine serum (FBS) and 1% penicillin–streptomycin at 37 °C in a humidified 5% CO₂ incubator. For induction of p16^INK4a^ expression, cells were seeded into 10 mm dishes and allowed to adhere overnight. Sub-confluent cultures were exposed to doxorubicin (Dox, 250 nmol/L) for 24 h. After drug removal, cultures were maintained in fresh DMEM for an additional 72 h to establish p16^INK4a^ expression. To assess epigenetic regulation, p16^INK4a+^ NIH-3T3 cells were treated with the histone demethylase inhibitor GSK-J4 (10 μmol/L) or the EZH2 inhibitor GSK-126 (5 μmol/L) for 48 h. Untreated cells served as controls. For immunofluorescence staining, cells were rinsed twice with PBS, fixed in 4% paraformaldehyde for 15 min at room temperature, and permeabilized with 0.3% Triton X-100 for 10 min. After blocking with 5% bovine serum albumin (BSA) for 1 h, samples were incubated overnight at 4 °C with primary antibodies against p16 (Abcam, ab26696) and Ki67 (Abcam, ab16667). After PBS washes, cells were incubated with Alexa Fluor® 488- or 594-conjugated secondary antibodies (Jackson ImmunoResearch) for 1 h in the dark. Nuclei were counterstained with DAPI (Vector Laboratories, H-1200) and mounted with antifade reagent. Fluorescent images were acquired using an inverted fluorescence microscope, and staining intensity was quantified using ImageJ software (NIH).

### Flow cytometry and FACS sorting of p16^INK4a+^ cells

At designated time points post-injury, the injured tendons were harvested, minced into small fragments, and digested with 0.2% collagenase type I (Sigma-Aldrich) and 0.1% dispase (Gibco) in DMEM at 37 °C for 1 h with gentle agitation. The digested tissue was passed through a 70 μm cell strainer, and the filtrate was washed twice with PBS containing 2% fetal bovine serum (FBS). p16-tdTOM^+^ cells were analyzed using a BD LSRII flow cytometer. For FACS sorting of p16^INK4a+^ mesenchymal cells, single-cell suspensions were incubated with anti–PDGFRα-BV421 antibody (Cat. 135923, BioLegend) for 30 min on ice. After washing, cells were analyzed and sorted using a BD FACSAria II flow cytometer. Flow cytometric data were processed using FlowJo software (version 10.8.1).

### Western blot analysis

Western blotting was performed as previously described with minor modifications.^49,50^ Briefly, Achilles tendons were collected at the indicated time points post-injury, and surrounding tissues were carefully removed under a stereomicroscope. For each sample, three tendons were pooled, crushed in liquid nitrogen, and homogenized in ice-cold RIPA buffer (Beyotime, P0013B) supplemented with protease and phosphatase inhibitors (Thermo Fisher Scientific). Lysates were incubated on ice for 30 min and centrifuged at 12 000 × *g* for 10 min at 4 °C. Protein concentrations were determined using the BCA assay (Thermo Fisher Scientific). Equal amounts of protein (30–50 μg) were separated by SDS–PAGE (10%–12%) and transferred onto PVDF membranes (Bio-Rad). Membranes were blocked in 5% nonfat milk for 1 h at room temperature and then incubated overnight at 4 °C with primary antibodies against JMJD3 (Abcam, ab38113), EZH2 (Cell Signaling Technology, 5246), UTX (Cell Signaling Technology, 33510), H3K27me3 (Cell Signaling Technology, 9733), COL1A1 (Cell Signaling Technology, 72026), COL3A1 (Proteintech, 22734-1-AP), β-Actin (Abcam, ab179467), and H3 (Abcam, ab52946). After washing, membranes were incubated with HRP-conjugated secondary antibodies (Jackson ImmunoResearch) for 1 h at room temperature. Bands were visualized using an enhanced chemiluminescence substrate (ECL, Amersham Biosciences) and imaged with a ChemiDoc™ XRS+ System (Bio-Rad). Band intensities were quantified using ImageJ (NIH), and protein levels were normalized to β-Actin as a loading control.

### qRT–PCR

Cells were collected in RNase-free tubes containing lysis buffer from the RNA extraction kit.^51^ Total RNA was extracted using the RNeasy Micro Kit (Qiagen) following the manufacturer’s protocol, and RNA quality and concentration were assessed using a NanoDrop 2000 spectrophotometer (Thermo Fisher Scientific). Complementary DNA (cDNA) was synthesized from 200 ng total RNA using the PrimeScript RT Reagent Kit (Takara). Quantitative PCR was performed with TB Green Premix Ex Taq II (Takara) on a CFX96 Real-Time PCR System (Bio-Rad). Relative gene expression was calculated using the 2^-ΔΔCt^ method, with β-actin serving as the internal control. Primer sequences for all target genes are listed in Table S2. Each reaction was run in triplicate, and data were obtained from at least three independent biological replicates.

### Single-cell transcriptome sequencing and bioinformatics analysis

Single-cell transcriptomic data were obtained from the GEO database (ID: PRJNA976191; PRJNA975881).^23,52^ Raw single-cell sequencing data was processed using SeekSoulTools to generate gene-cell expression matrices. Then analyses were performed using Seurat (4.1.1). Stringent quality control parameters were applied to eliminate low-quality cells based on the following criteria: number of detected genes (nFeature_RNA) within [100, 30 000], total UMI counts (nCount_RNA) within [300, 100 000]. Cell identities were assigned based on canonical markers and the SeekGene public database. Pathway enrichment was assessed using MSigDB and the GO gene set database.

### Statistics

Data are expressed as the mean ± standard deviation (SD). Comparisons between two groups were performed using unpaired, two-tailed Student’s *t*-tests. For analyses involving more than two groups, one-way or two-way analysis of variance (ANOVA) followed by Bonferroni’s post hoc test was applied. All datasets were tested for normal distribution and homogeneity of variance before statistical analysis. Statistical analyses were conducted using GraphPad Prism version 7.04 (GraphPad Software, San Diego, CA, USA). A *P* < 0.05 was considered statistically significant.

## Supplementary information

Supplementary Materials

## Data Availability

ScRNA-seq dataset PRJNA976191 and PRJNA975881 for mouse and human tendon injury, respectively, were used for analysis and are publicly available. All data supporting the findings of this study are included in the article and its Supplementary Information files. Additional data is available from the corresponding author upon reasonable request.

## References

[1] Nourissat, G., Berenbaum, F. & Duprez, D. Tendon injury: from biology to tendon repair. *Nat. Rev. Rheumatol.* **11**, 223–233 (2015). 25734975 10.1038/nrrheum.2015.26

[2] Darrieutort-Laffite, C., Blanchard, F., Soslowsky, L. J. & Le Goff, B. Biology and physiology of tendon healing. *Jt. Bone Spine* **91**, 105696 (2024). 10.1016/j.jbspin.2024.105696 38307405

[3] Titan, A. L. & Longaker, M. T. A fine balance in tendon healing. *Nat. Cell Biol.* **21**, 1466–1467 (2019). 31768049 10.1038/s41556-019-0432-0

[4] Schulze-Tanzil, G. G., Delgado-Calcares, M., Stange, R., Wildemann, B. & Docheva, D. Tendon healing: a concise review on cellular and molecular mechanisms with a particular focus on the Achilles tendon. *Bone Jt. Res.* **11**, 561–574 (2022). 10.1302/2046-3758.118.BJR-2021-0576.R1 PMC9396922 35920195

[5] Park, S. S. et al. Distribution and impact of p16^INK4A+^ senescent cells in elderly tissues: a focus on senescent immune cell and epithelial dysfunction. *Exp. Mol. Med.* **56**, 2631–2641 (2024). 39617789 10.1038/s12276-024-01354-4 PMC11671602

[6] Pellarin, I. et al. Cyclin-dependent protein kinases and cell cycle regulation in biology and disease. *Signal Transduct. Target. Ther.* **10**, 11 (2025). 39800748 10.1038/s41392-024-02080-z PMC11734941

[7] Bi, Y. et al. Exosomal miR-302b rejuvenates aging mice by reversing the proliferative arrest of senescent cells. *Cell Metab.* **37**, 527–541 (2025). 39818209 10.1016/j.cmet.2024.11.013

[8] Dong, T. et al. Calumenin prevents fibroblast senescence and lung aging by promoting vimentin proteostasis. *Proc. Natl. Acad. Sci. USA* **123**, e1868244173 (2026). 10.1073/pnas.2426723123 PMC12849709 41557802

[9] Zhao, H. et al. Identifying specific functional roles for senescence across cell types. *Cell* **187**, 7314–7334 (2024). 39368477 10.1016/j.cell.2024.09.021

[10] Tu, C. et al. Targeting the chromatin remodeler BAZ2B mitigates hepatic senescence and MASH fibrosis. *Nat. Aging* **5**, 1063–1078 (2025). 40389730 10.1038/s43587-025-00862-w

[11] Demaria, M. et al. An essential role for senescent cells in optimal wound healing through secretion of PDGF-AA. *Dev. Cell.* **31**, 722–733 (2014). 25499914 10.1016/j.devcel.2014.11.012 PMC4349629

[12] Reyes, N. S. et al. Sentinel p16^INK4a+^ cells in the basement membrane form a reparative niche in the lung. *Science* **378**, 192–201 (2022). 36227993 10.1126/science.abf3326 PMC10621323

[13] Holoch, D. & Moazed, D. RNA-mediated epigenetic regulation of gene expression. *Nat. Rev. Genet.* **16**, 71–84 (2015). 25554358 10.1038/nrg3863 PMC4376354

[14] Pashos, A. R. S. et al. H3K36 methylation regulates cell plasticity and regeneration in the intestinal epithelium. *Nat. Cell Biol.* **27**, 202–217 (2025). 39779942 10.1038/s41556-024-01580-y PMC12342706

[15] Crouch, J., Shvedova, M., Thanapaul, R. J. R. S., Botchkarev, V. & Roh, D. Epigenetic regulation of cellular senescence. *Cells* **11**, 672 (2022). 35203320 10.3390/cells11040672 PMC8870565

[16] Allas, L. et al. JMJD3 and UTX as key targets for gene-modified mesenchymal stem cell therapy in cartilage tissue engineering. *Mol. Ther.* **34**, S1516–S1525 (2026). 10.1016/j.ymthe.2026.01.018 PMC13154314 41566779

[17] Chen, Y. et al. The EZH2-H3K27me3 axis modulates aberrant transcription and apoptosis in cyclophosphamide-induced ovarian granulosa cell injury. *Cell Death Discov.* **9**, 413 (2023). 37963880 10.1038/s41420-023-01705-6 PMC10646043

[18] Ding, Y. et al. JMJD3: a critical epigenetic regulator in stem cell fate. *Cell Commun. Signal.* **19**, 72 (2021). 34217316 10.1186/s12964-021-00753-8 PMC8254972

[19] Manni, W., Jianxin, X., Weiqi, H., Siyuan, C. & Huashan, S. JMJD family proteins in cancer and inflammation. *Signal Transduct. Target. Ther.* **7**, 304 (2022). 36050314 10.1038/s41392-022-01145-1 PMC9434538

[20] Kochat, V. et al. JMJD3 aids in reprogramming of bone marrow progenitor cells to hepatic phenotype through epigenetic activation of hepatic transcription factors. *PLoS ONE* **12**, e0173977 (2017). 28328977 10.1371/journal.pone.0173977 PMC5362104

[21] Shan, Y. et al. JMJD3 and UTX determine fidelity and lineage specification of human neural progenitor cells. *Nat. Commun.* **11**, 382 (2020). 31959746 10.1038/s41467-019-14028-x PMC6971254

[22] Cherief, M. et al. TrkA-mediated sensory innervation of injured mouse tendon supports tendon sheath progenitor cell expansion and tendon repair. *Sci. Transl. Med.* **15**, eade4619 (2023). 38117901 10.1126/scitranslmed.ade4619

[23] Zhang, X. et al. Multi-omics analysis of human tendon adhesion reveals that ACKR1-regulated macrophage migration is involved in regeneration. *Bone Res.* **12**, 27 (2024). 38714649 10.1038/s41413-024-00324-w PMC11076548

[24] Xie, H. et al. PDGF-BB secreted by preosteoclasts induces angiogenesis during coupling with osteogenesis. *Nat. Med.* **20**, 1270–1278 (2014). 25282358 10.1038/nm.3668 PMC4224644

[25] Rafii, S., Butler, J. M. & Ding, B. Angiocrine functions of organ-specific endothelial cells. *Nature* **529**, 316–325 (2016). 26791722 10.1038/nature17040 PMC4878406

[26] Lu, Y. et al. CGRP sensory neurons promote tissue healing via neutrophils and macrophages. *Nature* **628**, 604–611 (2024). 38538784 10.1038/s41586-024-07237-y PMC11023938

[27] Iyer, S. S. Epigenetic regulation of bone healing: implications for fracture repair and clinical treatment strategies. *Yale J. Biol. Med.* **98**, 159–170 (2025). 40589933 10.59249/HSYL8000 PMC12204031

[28] Rouault, C. D., Charafe-Jauffret, E. & Ginestier, C. The interplay of DNA damage, epigenetics and tumour heterogeneity in driving cancer cell fitness. *Nat. Commun.* **16**, 8733 (2025). 41028732 10.1038/s41467-025-64445-4 PMC12484684

[29] Shen, X. et al. EZH1 mediates methylation on histone H3 lysine 27 and complements EZH2 in maintaining stem cell identity and executing pluripotency. *Mol. Cell.* **32**, 491–502 (2008). 19026780 10.1016/j.molcel.2008.10.016 PMC2630502

[30] Nakka, K. et al. JMJD3 activated hyaluronan synthesis drives muscle regeneration in an inflammatory environment. *Science* **377**, 666–669 (2022). 35926054 10.1126/science.abm9735

[31] Zhou, T. et al. GSK-126 attenuates cell apoptosis in ischemic brain injury by modulating the EZH2-H3K27me3-Bcl2l1 axis. *Mol. Neurobiol.* **61**, 3369–3383 (2024). 37989985 10.1007/s12035-023-03808-8

[32] Dalpatraj, N., Naik, A. & Thakur, N. GSK-J4: An H3K27 histone demethylase inhibitor, as a potential anti-cancer agent. *Int. J. Cancer* **153**, 1130–1138 (2023). 37165737 10.1002/ijc.34559

[33] Muñoz-Espín, D. & Serrano, M. Cellular senescence: from physiology to pathology. *Nat. Rev. Mol. Cell Biol.* **15**, 482–496 (2014). 24954210 10.1038/nrm3823

[34] Childs, B. G. et al. Senescent cells: an emerging target for diseases of ageing. *Nat. Rev. Drug Discov.* **16**, 718–735 (2017). 28729727 10.1038/nrd.2017.116 PMC5942225

[35] Zhang, F. et al. Histone demethylase JMJD3 is required for osteoblast differentiation in mice. *Sci. Rep.* **5**, 13418 (2015). 26302868 10.1038/srep13418 PMC4548232

[36] Gao, Y., Wang, N. & Jia, D. H3K27 tri-demethylase JMJD3 inhibits macrophage apoptosis by promoting ADORA2A in lipopolysaccharide-induced acute lung injury. *Cell Death Discov.* **8**, 475 (2022). 36456564 10.1038/s41420-022-01268-y PMC9715944

[37] Yang, D., Okamura, H., Nakashima, Y. & Haneji, T. Histone demethylase Jmjd3 regulates osteoblast differentiation via transcription factors Runx2 and osterix. *J. Biol. Chem.* **288**, 33530–33541 (2013). 24106268 10.1074/jbc.M113.497040 PMC3837102

[38] Agger, K. et al. UTX and JMJD3 are histone H3K27 demethylases involved in HOX gene regulation and development. *Nature* **449**, 731–734 (2007). 17713478 10.1038/nature06145

[39] Ming-Chin Lee, K. et al. Type I interferon antagonism of the JMJD3-IRF4 pathway modulates macrophage activation and polarization. *Cell Rep.* **39**, 110719 (2022). 35443173 10.1016/j.celrep.2022.110719

[40] Davis, F. M. et al. Inhibition of macrophage histone demethylase JMJD3 protects against abdominal aortic aneurysms. *J. Exp. Med.* **218**, e20201839 (2021). 33779682 10.1084/jem.20201839 PMC8008365

[41] Doñas, C. et al. The histone demethylase inhibitor GSK-J4 limits inflammation through the induction of a tolerogenic phenotype on DCs. *J. Autoimmun.* **75**, 105–117 (2016). 27528513 10.1016/j.jaut.2016.07.011

[42] Zhen, G. et al. Mechanical stress determines the configuration of TGFβ activation in articular cartilage. *Nat. Commun.* **12**, 1706 (2021). 33731712 10.1038/s41467-021-21948-0 PMC7969741

[43] Li, Y. et al. Nociceptive sensory neuron-derived NGF orchestrates a fibrotic mesenchymal stromal cell neurogenic niche to drive tendon pathological fibrosis. *Nat. Commun.* **17**, 650 (2025). 41365914 10.1038/s41467-025-67396-y PMC12816624

[44] Cook, A. D. et al. TNF and granulocyte macrophage-colony stimulating factor interdependence mediates inflammation via CCL17. *JCI Insight* **3**, e99249 (2018). 29563337 10.1172/jci.insight.99249 PMC5926922

[45] Pan, Y. et al. GSKJ4 protects mice against early sepsis via reducing proinflammatory factors and up-regulating MiR-146a. *Front. Immunol.* **9**, 2272 (2018). 30337925 10.3389/fimmu.2018.02272 PMC6179039

[46] Zhou, T. et al. Enhancer of zeste homolog 2-catalysed H3K27 trimethylation plays a key role in acute-on-chronic liver failure via TNF-mediated pathway. *Cell Death Dis.* **9**, 590 (2018). 29789597 10.1038/s41419-018-0670-2 PMC5964223

[47] Chaudhary, P., Guragain, D., Chang, J. & Kim, J. TPH1 and 5-HT(7) receptor overexpression leading to gemcitabine-resistance requires non-canonical permissive action of EZH2 in pancreatic ductal adenocarcinoma. *Cancers* **13**, 5305 (2021). 34771469 10.3390/cancers13215305 PMC8582390

[48] Lake, S. P. et al. Guidelines for ex vivo mechanical testing of tendon. *J. Orthop. Res.* **41**, 2105–2113 (2023). 37312619 10.1002/jor.25647 PMC10528429

[49] Liu, X. et al. Osteoclasts protect bone blood vessels against senescence through the angiogenin/plexin-B2 axis. *Nat. Commun.* **12**, 1832 (2021). 33758201 10.1038/s41467-021-22131-1 PMC7987975

[50] Liu, X. et al. Oxylipin-PPARγ-initiated adipocyte senescence propagates secondary senescence in the bone marrow. *Cell Metab.* **35**, 667–684 (2023). 37019080 10.1016/j.cmet.2023.03.005 PMC10127143

[51] Zhang, P. et al. 3D-Printed strontium silicate hydrogel scaffold promotes sensory innervation and tissue regeneration through modulating macrophage senescence. *Adv. Funct. Mater* **19**, e29224 (2026).

[52] Zhang, X. et al. Myeloid cells and sensory nerves mediate peritendinous adhesion formation via prostaglandin E2. *Adv. Sci.* **11**, e2405367 (2024). 10.1002/advs.202405367 PMC11516151 39207041

