# Supplementary material for: JMJD3-driven epigenetic reprogramming of p16INK4a-positive cells promotes tendon regeneration
Source: Bone Res. 2026 Jun 11;14:65. doi: 10.1038/s41413-026-00537-1 (PMC13261133; doi:10.1038/s41413-026-00537-1)
Supplement: Supplementary file 1 — Supplementary Materials [file 41413_2026_537_MOESM1_ESM.docx]

**
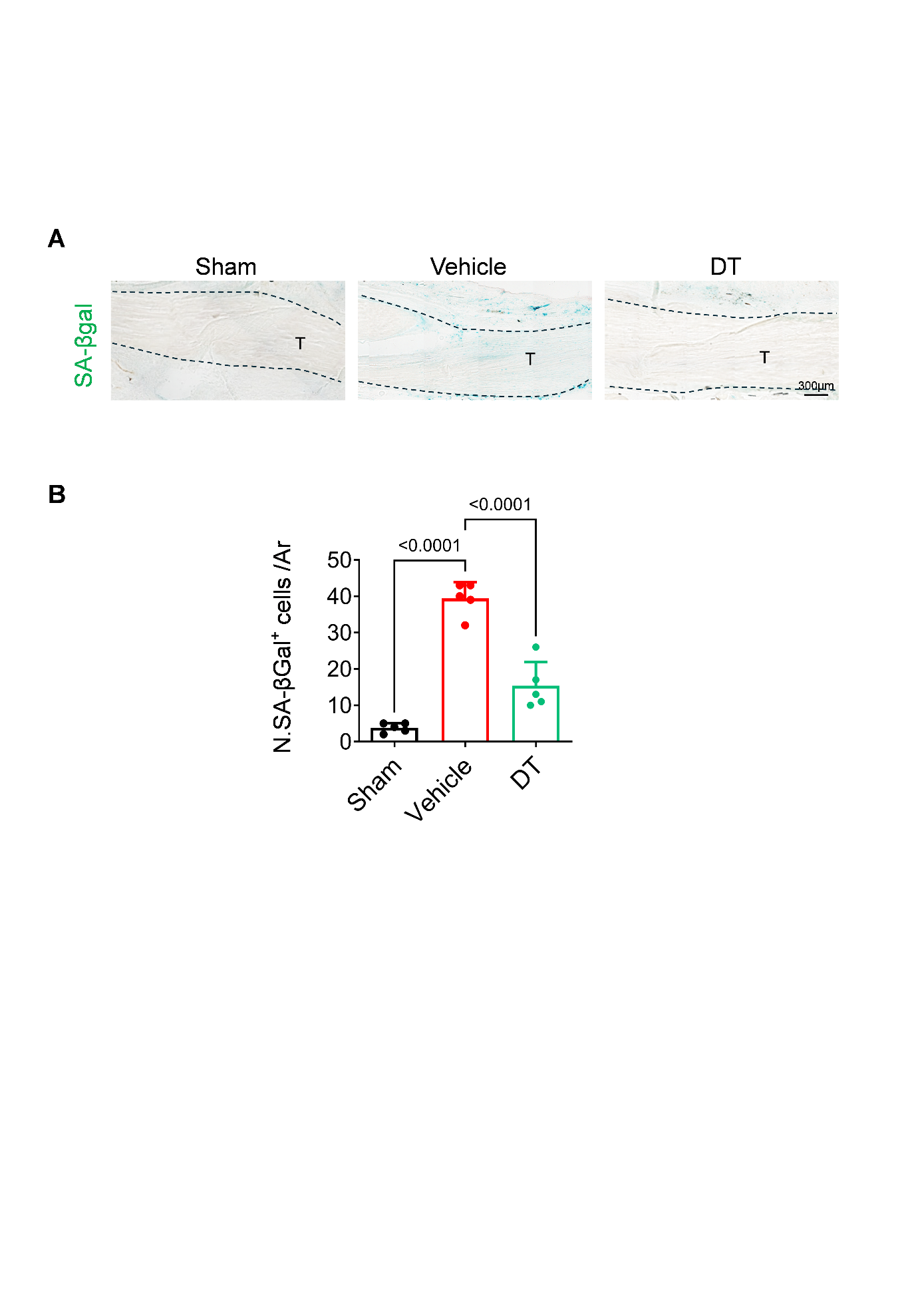
**

**Supplementary Figure 1 SA-β-gal Staining in Tendon Tissue in *p16-iDTR* Mice.**

(**A**) Representative images of SA-βGal staining in tendon sections from control, vehicle-treated, and diphtheria toxin (DT)–treated groups. T, tendon area. (**B**) Quantification of SA-βGal⁺ cells per mm² of tendon tissue area among the indicated groups. n = 5–6 mice per group. Data are presented as mean ± SD. Statistical significance was determined using one-way ANOVA.

**
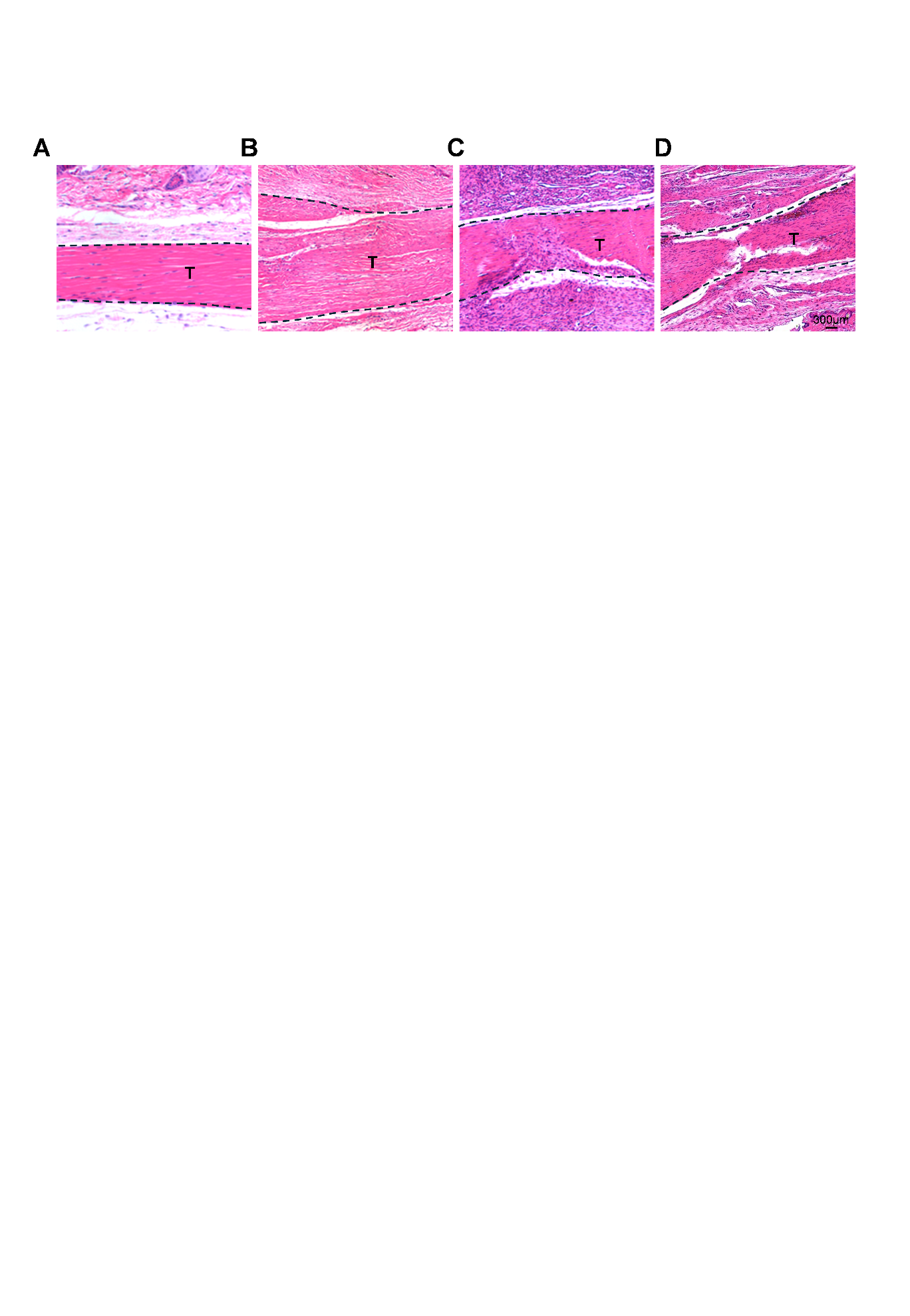
**

**Supplementary Figure** **2 Healing Grading Scale Assessment of tendon repair.**

(**A**–**D**) Representative histological images illustrating the tendon healing grading criteria based on collagen fiber organization, epitenon integrity, and overall tissue morphology. (**A**) Grade 1 (Excellent): Continuous tendon structure with well-organized, parallel collagen bundles and an intact epitenon. (**B**) Grade 2 (Good): Collagen bundles exhibit near-normal alignment with mild fibrosis or partial disruption of the epitenon. (**C**) Grade 3 (Fair): Collagen fibers display irregular organization with partial discontinuity and infiltration of granulation tissue. (**D**) Grade 4 (Poor): Severely impaired tendon healing characterized by marked disorganization of collagen fibers and extensive granulation tissue formation.

**
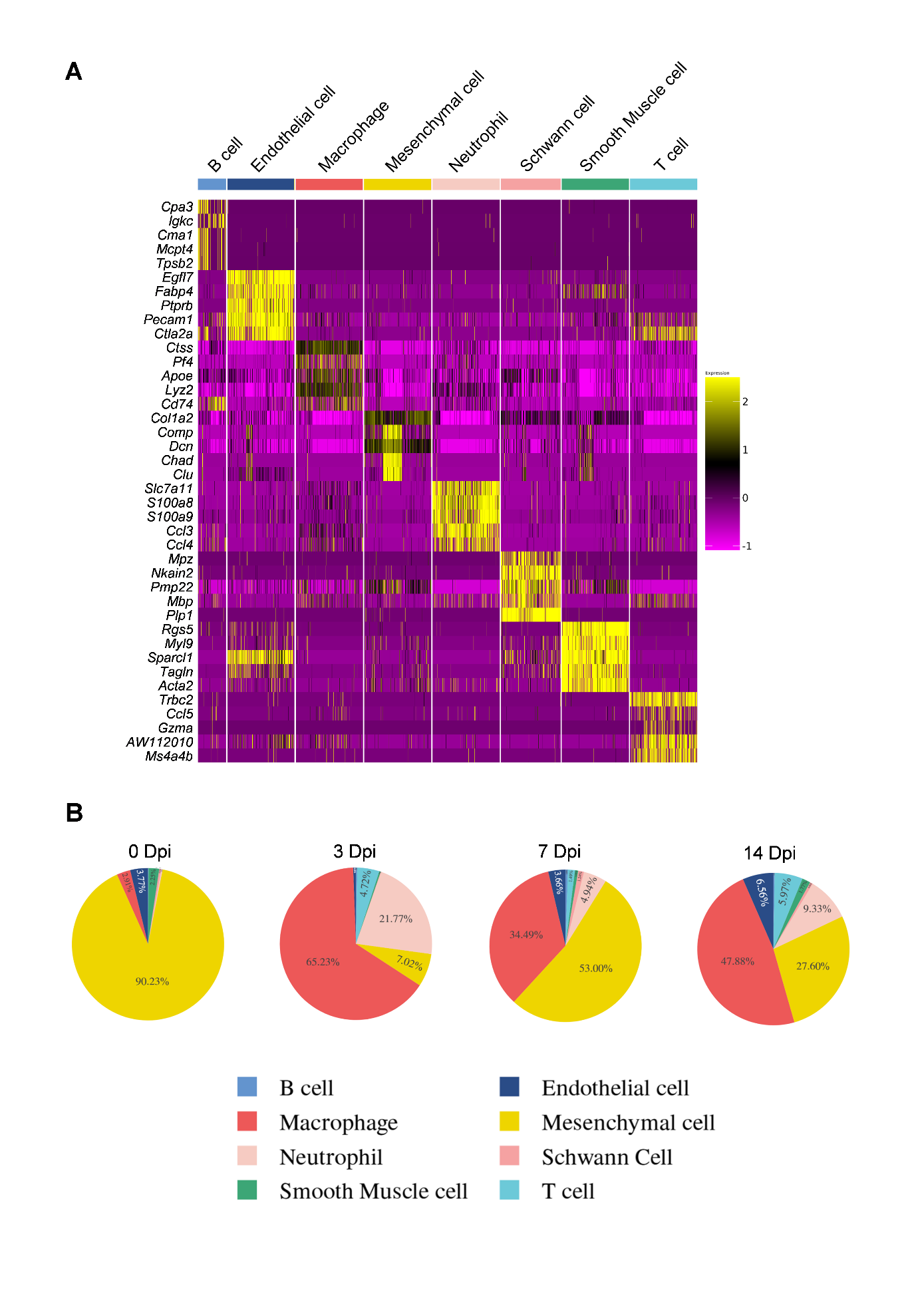
**

**Supplementary Figure 3 Cell-type Identification and Dynamic Composition Changes During Tendon Repair.**

(**A**) Heatmap showing the expression of representative marker genes used to identify major cell populations in single-cell sequencing, including B cells, endothelial cells, macrophages, neutrophils, Schwann cells, smooth muscle cells, T cells, and mesenchymal cells. Each column represents a single cell, and color intensity indicates normalized gene expression levels. (**B**) Pie charts showing the relative proportions of major cell populations in uninjured tendons at 0, 3, 7, and 14 Dpi.

**
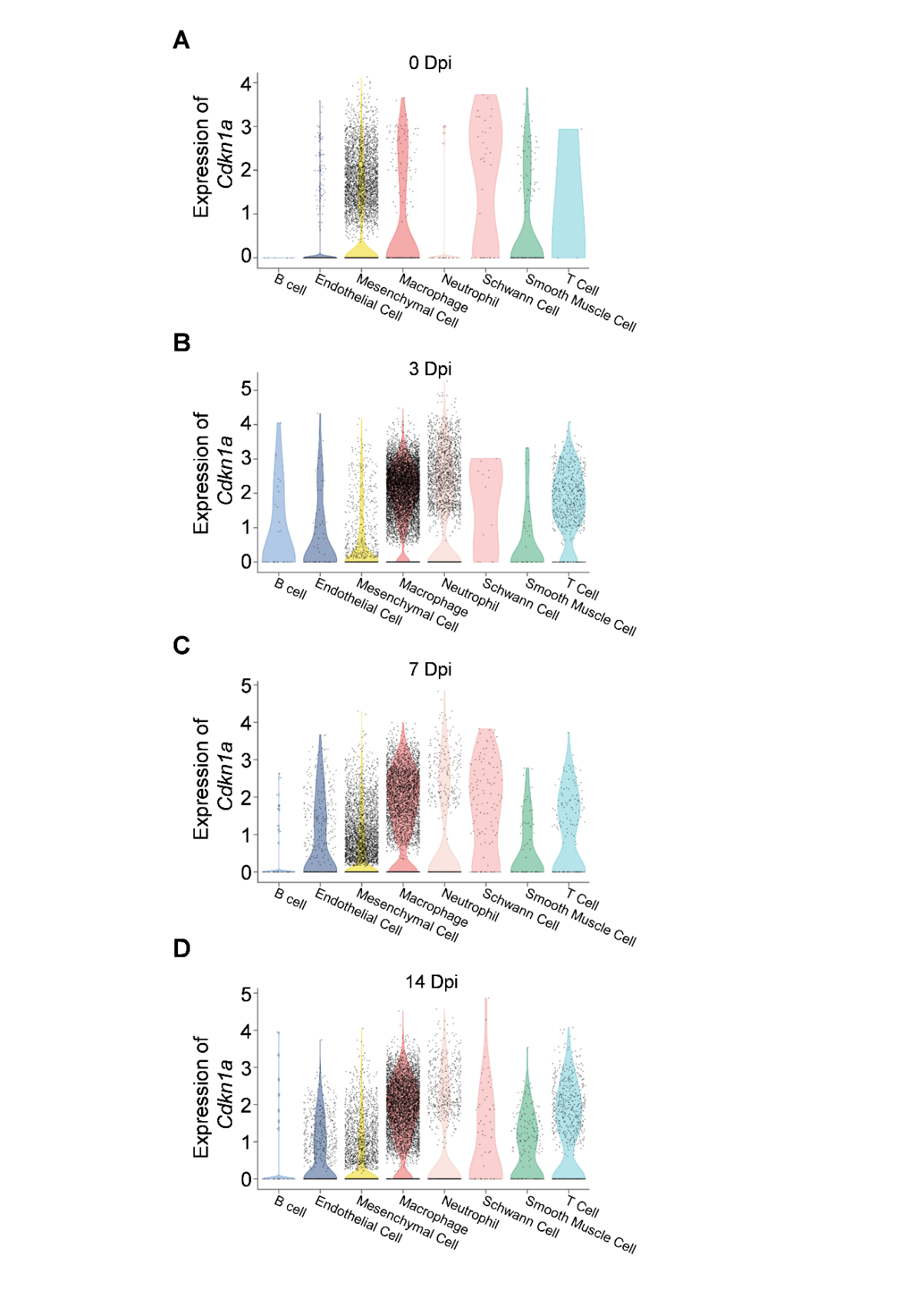
**

**Supplementary Figure 4** **Expression Level of *Cdkn1a* During Tendon Repair.**

(**A**-**D**) Violin plots illustrating gene expression levels of *Cdkn1a* across cell types at 0, 3, 7, and 14 Dpi.


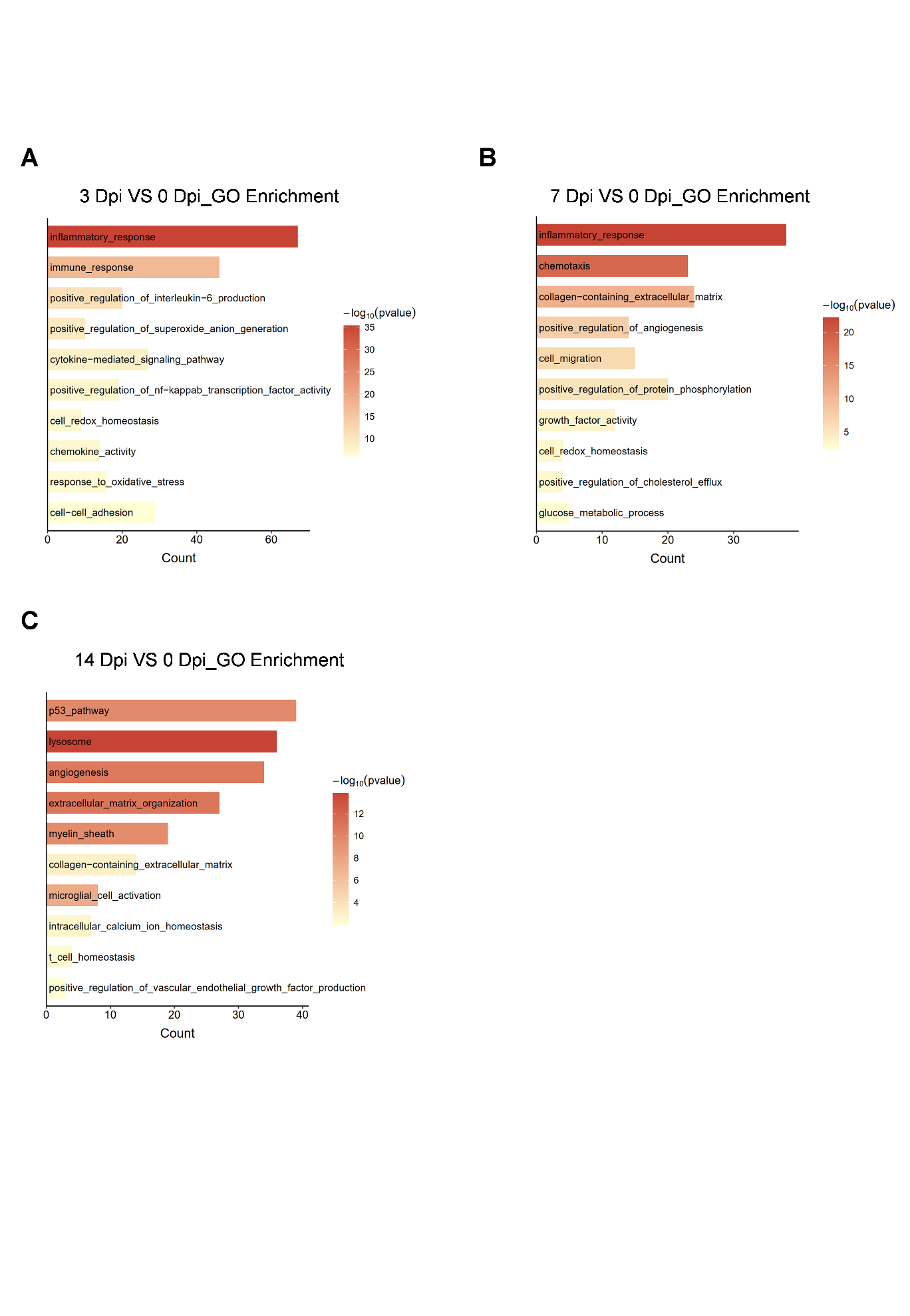


**Supplementary Figure 5. Gene Ontology Pathway Enrichment Analysis of Differentially Expressed Genes During Tendon Repair.**

(**A**–**C**) Gene Ontology (GO) pathway enrichment analysis of DEGs at 3, 7, and 14 Dpi compared with uninjured tendons. (**A**) Enriched biological processes at 3 Dpi, showing significant enrichment of inflammatory response–related pathways. (**B**) Enriched biological processes at 7 Dpi, highlighting pathways associated with collagen-containing extracellular matrix organization, angiogenesis, and cell migration. (**C**) Enriched biological processes at 14 Dpi, showing enrichment of p53 signaling pathway, lysosome-related processes, angiogenesis, and extracellular matrix organization. Bar length represents the number of genes associated with each GO term, and color intensity indicates statistical significance based on −log₁₀ (p value).


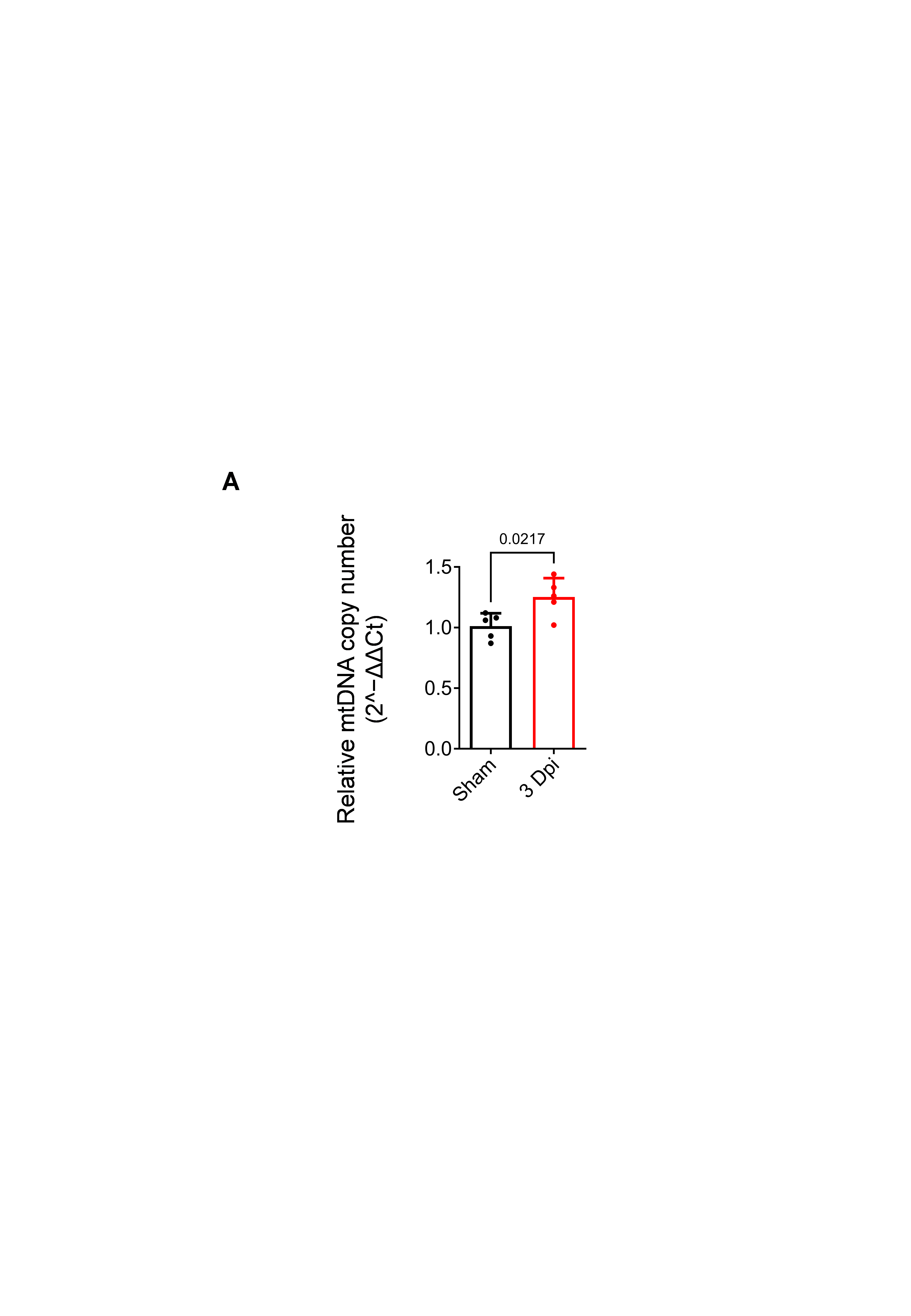


**Supplementary Figure 6. Increased Mitochondrial DNA Copy Number During Early Tendon Repair.**

(**A**) Quantitative PCR analysis showing relative mitochondrial DNA (mtDNA) copy number in tendon tissue at 0 and 3 Dpi, normalized using the 2⁻ΔΔCt method. n = 5 mice per group. Data are presented as mean ± SD. Statistical significance was determined using an unpaired two-tailed Student’s t test.


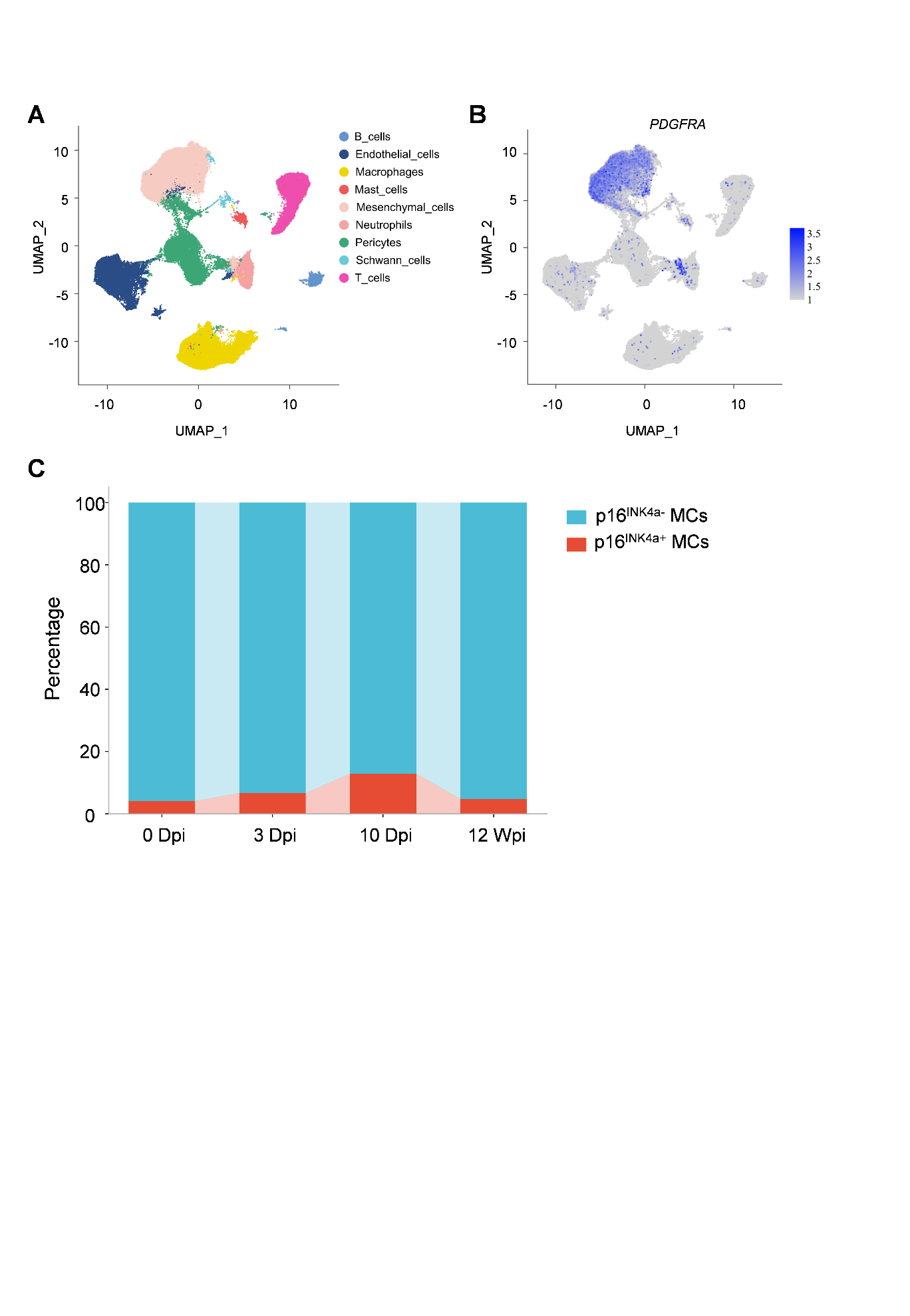


**Supplementary Figure 7. Single-cell RNA Sequencing Analysis of p16^INK4a^⁺ Mesenchymal Cells During Human Tendon Injury.**

(**A**) UMAP showing major cell populations identified from a publicly available human tendon injury single-cell RNA sequencing dataset (PRJNA975881). (**B**) Feature plot showing expression of *PDGFA*, a marker gene for mesenchymal cells, across all identified cell populations. (**C**) Proportion of p16^INK4a^⁺ and p16^INK4a^⁻ mesenchymal cells at different time points following tendon injury, including 0 Days, 3 Days, 10 Days, and 12 weeks post-injury.

**
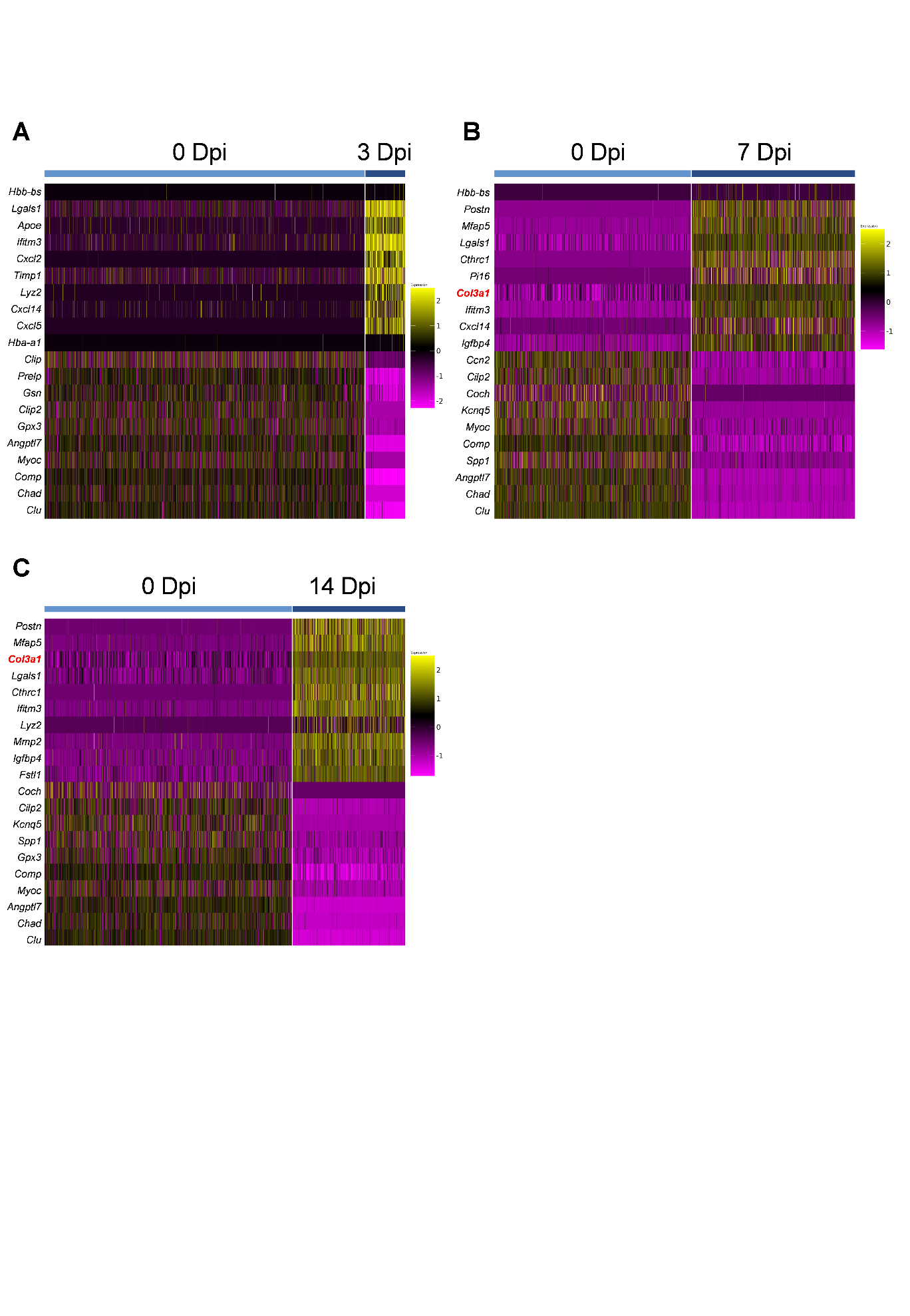
**

**Supplementary Figure 8 Differential Gene Expression Analysis of Mesenchymal Cells During Tendon Repair.**

(**A**–**C**) Heatmaps showing differential gene expression profiles of mesenchymal cells at 3, 7, and 14 Dpi compared with 0 Dpi. Each column represents an individual cell, and each row represents a gene. Color intensity indicates normalized gene expression levels.


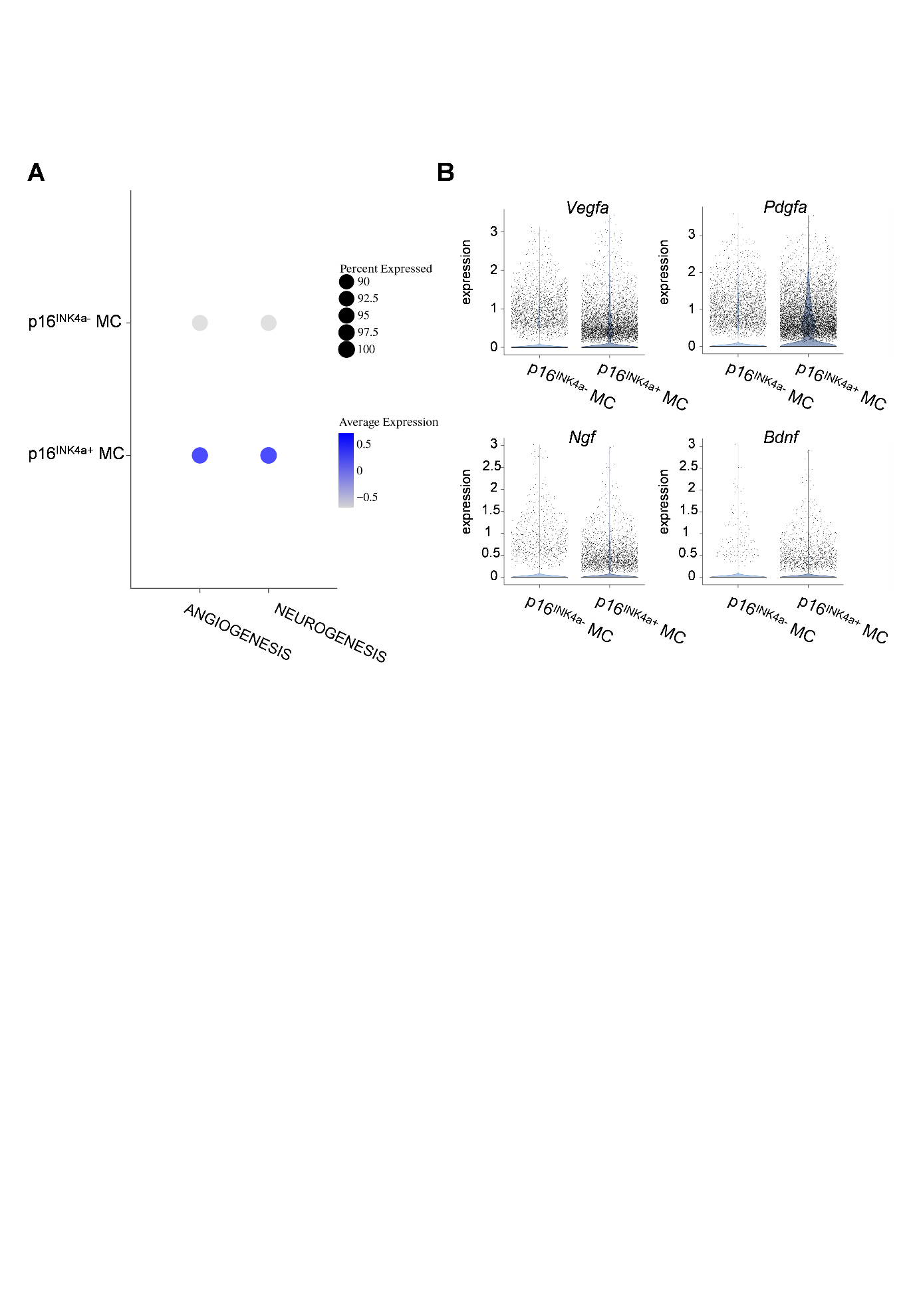


**Supplementary Figure 9. Angiogenic and Neurotrophic Gene Expression in p16^INK4a^⁺ Mesenchymal Cells During Tendon Repair.**

(**A**) Dot plot showing enrichment of angiogenesis and neurogenesis-related pathways in p16^INK4a^⁺ and p16^INK4a^⁻ mesenchymal cells based on single-cell RNA sequencing analysis. (**B**) Violin plots showing expression levels of pro-angiogenic genes *Vegfa* and *Pdgfa*, and neurotrophic genes *Ngf* and *Bdnf* in p16^INK4a^⁺ and p16^INK4a^⁻ mesenchymal cells.


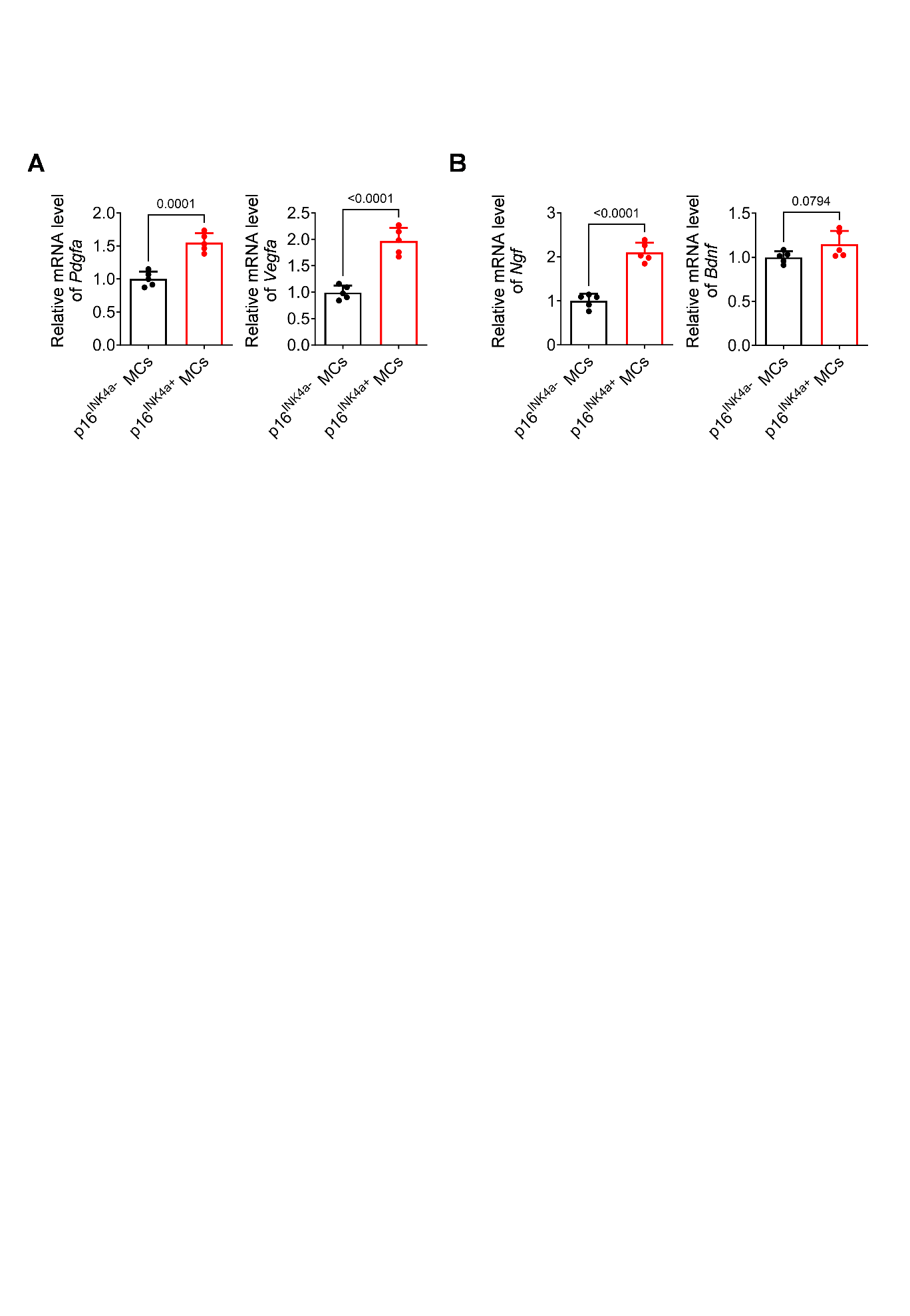


**Supplementary Figure 10. Increased Expression of Angiogenic and Neurotrophic Genes in p16^INK4a^⁺ Mesenchymal Cells.**

(**A**–**B**) Quantitative qRT–PCR analysis showing relative mRNA expression levels of pro-angiogenic genes *Pdgfa* and *Vegfa* (**A**), and neurotrophic genes *Ngf* and *Bdnf* (**B**) in p16^INK4a^⁺ and p16^INK4a^⁻ mesenchymal cells. n = 5 mice per group. Data are presented as mean ± SD. Statistical significance was determined using unpaired two-tailed Student’s t tests.


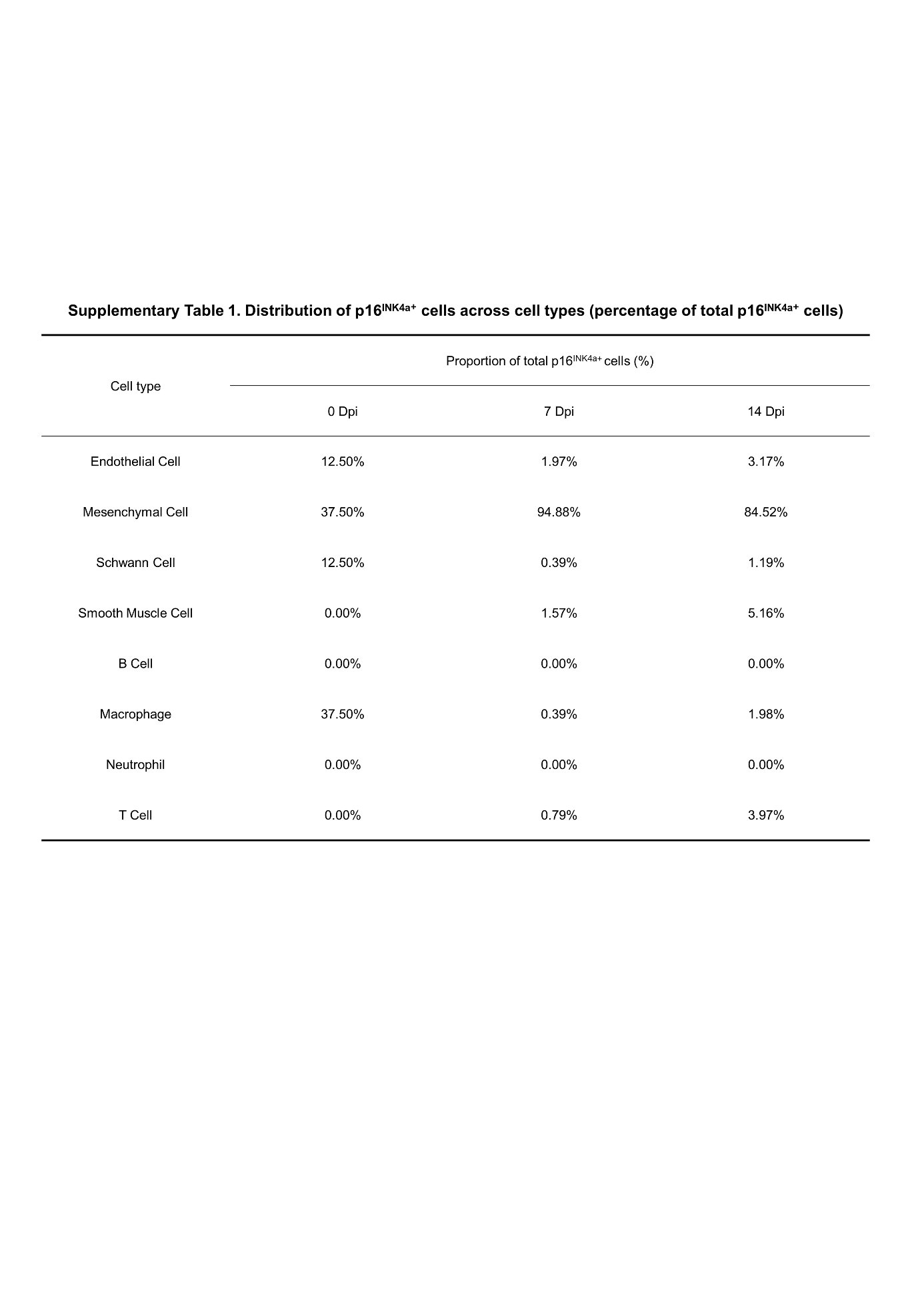


**Supplementary Table 1. Distribution of p16^INK4a+^ cells across major cell populations during tendon repair.**

Percentages indicate the proportion of total p16^INK4a+^ cells assigned to each cell type at 0, 7, and 14 Dpi, based on single-cell RNA sequencing analysis. Mesenchymal cells represent the dominant p16^INK4a+^ population during the reparative phase.


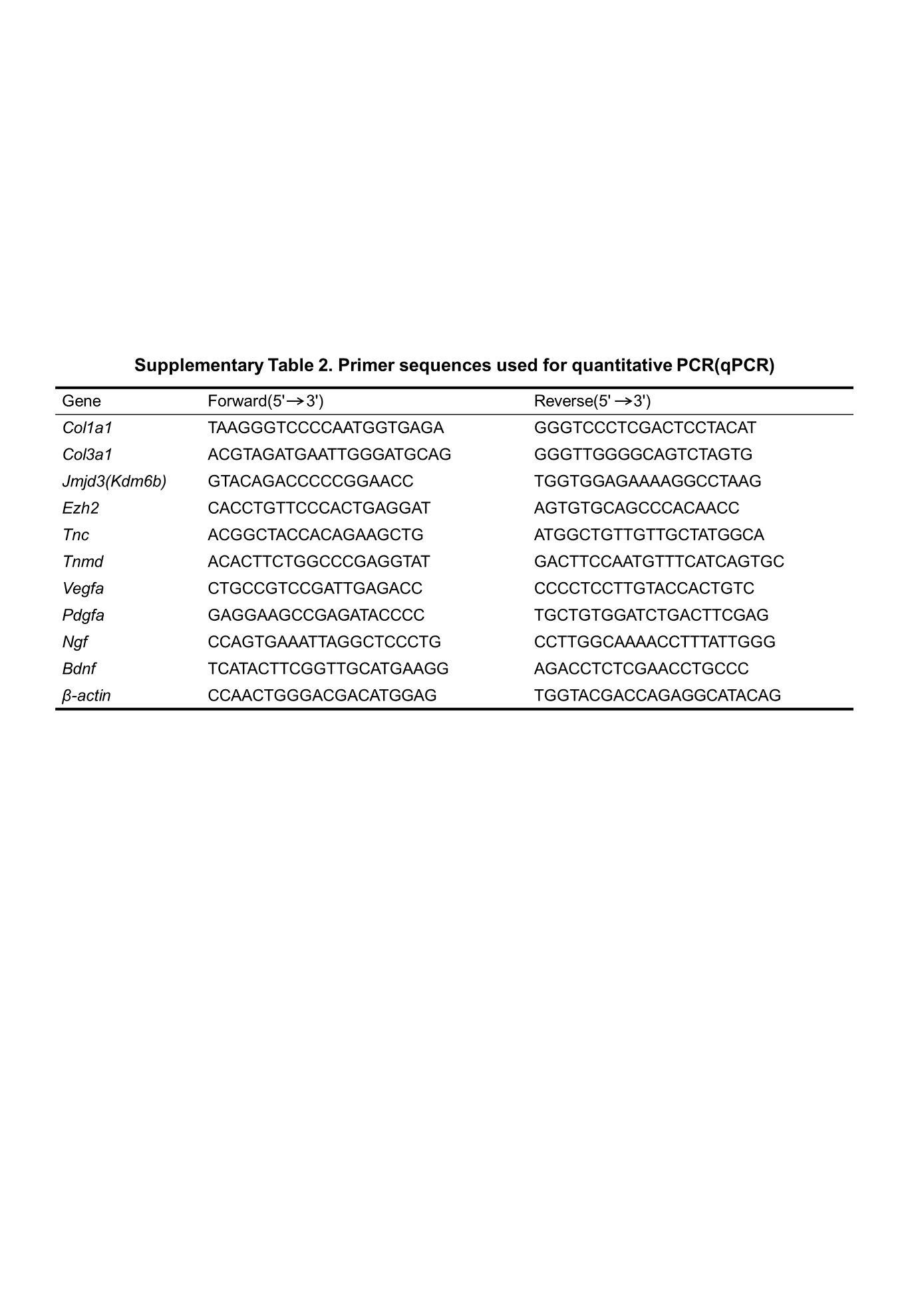


**Supplementary Table 2 Primer Sequences Used for qPCR.** Primer sequences for mouse genes analyzed by qPCR. All sequences are listed in the 5′→3′ direction.
